# Effect of intravenous vitamin C on adult septic patients: a systematic review and meta-analysis

**DOI:** 10.3389/fnut.2023.1211194

**Published:** 2023-08-03

**Authors:** Huoyan Liang, Qingqing Mu, Wenju Sun, Liming Liu, Simin Qiu, Zili Xu, Yuqing Cui, Yan Yan, Tongwen Sun

**Affiliations:** ^1^General Intensive Care Unit, Henan Key Laboratory of Critical Care Medicine, Zhengzhou Key Laboratory of Sepsis, Henan Engineering Research Center for Critical Care Medicine, The First Affiliated Hospital of Zhengzhou University, Zhengzhou, China; ^2^Academy of Medical Sciences, Zhengzhou University, Zhengzhou, China; ^3^Academy of Clinical Medicine, Zhengzhou University, Zhengzhou, China

**Keywords:** vitamin C, sepsis, septic shock, hydrocortisone, meta-analysis

## Abstract

**Background:**

An increasing number of studies indicate that vitamin C (VC) reduces the mortality of adult septic patients, while some articles suggest otherwise. We performed this systematic review and meta-analysis to resolve the discrepancies in reported results concerning the efficacy of VC in septic patients.

**Methods:**

We comprehensively searched MEDLINE, EMBASE, and the Cochrane Central Register of Controlled trials for randomized controlled trials (RCTs) evaluating the efficacy of intravenous VC (IVVC) on adult septic patients published from inception to November 28, 2022. The quality of outcomes for eligible studies was assessed using the Recommendations Assessment, Development, and Evaluation methodology. The results were analyzed using the pooled mean difference (MD) or risk ratio (RR) and 95% confidence intervals (CIs).

**Results:**

Twenty-two studies (3,570 adult septic patients) were included. IVVC treatment did not improve 28-day mortality compared to the control group (RR, 0.92; 95% CI, 0.81–1.04; *I*^2^ = 26%; evidence risk, moderate). IVVC monotherapy decreased mortality (RR, 0.69; 95% CI, 0.52–0.93; *I*^2^ = 57%), whereas combination therapy did not affect mortality (RR, 1.03; 95% CI, 0.90–1.17; *I*^2^ =0%). IVVC had a trend to decrease the mortality of septic patients (RR, 0.83; 95% CI, 0.69–1.00; *I*^2^ = 33%) but did not affect septic shock patients (RR, 1.01; 95% CI, 0.85–1.21; *I*^2^ = 18%). IVVC reduced the duration of vasopressor use (MD, −8.45; 95% CI, −15.43 to −1.47; evidence risk, very low) but did not influence the incidence of AKI, ICU length of stay, duration of mechanical ventilation.

**Conclusions:**

IVVC treatment did not improve the 28-day mortality in septic patients. Subgroup analysis indicated that VC had a trend to decrease the 28-day mortality in patients with sepsis but not septic shock. IVVC monotherapy, rather than combination therapy, decreased the 28-day mortality in septic patients. The findings imply that Hydrocortisone, Ascorbic acid, Thiamine (HAT) combination therapy is not superior to IVVC monotherapy for septic patients. These findings warrant further confirmation in future studies, which should also investigate the mechanisms underlying the enhanced efficacy of IVVC monotherapy in septic patients.

**Systematic review registration:**

https://inplasy.com/.

## Introduction

Sepsis is a dysregulated host response to infection (1), and it is the leading cause of intensive care unit (ICU) mortality worldwide (2). Sepsis can result in multiple organ failure, placing a significant economic burden on healthcare systems (3). The primary treatments for sepsis include antimicrobial therapy, source control, and organ support.

In sepsis, the oxidative stress response is substantially enhanced, causing mitochondrial damage, which is a driving factor for sequential organ failure (4–6). Vitamin C (VC) is an anti-inflammatory and antioxidant agent that protects against reactive oxygen species (ROS)-induced damage to the epithelial barrier (7). The previous indicated that VC could interact with tocopherol, glutathione, and thioredoxin, and stimulate the biosynthesis and activation of catalase and glutathione peroxidase (8). VC can also increase the bioavailability of NO, potentially improving microcirculatory perfusion. The mechanism behind this is that VC prevents the oxidation of tetrahydrobiopterin (BH4), which maintains the coupled activity of endothelial NOS, preventing the production of superoxide and the aggravation of oxidative damage (9). Humans cannot synthesize VC, and its levels are low in many critically ill patients (10), making supplementation potentially beneficial.

Previous studies (11–14) have demonstrated that intravenous VC (IVVC) therapy could reduce oxidative stress levels and improve clinical outcomes. Furthermore, some randomized controlled trials (RCTs) have further confirmed the therapeutic efficacy of IVVC for sepsis compared to placebo (15–17). Meta-analyses have evaluated the effect of VC treatment either as combination therapy with thiamine or other agents (18–23) or as mono-intravenous VC therapy (24), often including retrospective data to obtain pooled results, which limits the interpretability of their findings (19, 24–29). Therefore, as the effect of VC on sepsis remains unclear, numerous RCTs have been further conducted to evaluate the effect of VC on sepsis (30–32). This study aimed to assess the effect of VC on septic patients to address the discrepancies in the results concerning the benefit of VC administration in sepsis.

## Methods

The protocol of this systematic review and meta-analysis was registered on INPLASY (INPLASY2022110147).

### Search strategy and study identification

We conducted this meta-analysis following the Preferred Reporting Items for Systematic Reviews and Meta-analyses (PRISMA) criteria. The three databases MEDLINE, EMBASE, and the Cochrane Central Register of Controlled trials were searched to identify eligible studies published from the inception of the databases to November 28, 2022. The keyword search terms were VC, critically ill patients, and sepsis (detailed search strategy in Supplementary Table 1).

### Inclusion criteria

We included RCTs that met the following criteria: (1) design: RCTs; (2) population: adult patients (≥18 years) with a diagnosis of sepsis or septic shock. The sequential organ failure assessment (SOFA) score of two points or more is defined as organ dysfunction; (3) intervention: septic patients treated with VC; (4) control group: no VC administration; (5) reported outcomes: at least one of the following 28-day mortality, risk of incidence of acute kidney injury (AKI) after enrolment, intensive care unit length of stay (ICU-LOS), change in SOFA score at day 3, 4, or 5 from baseline, and duration of mechanical ventilation (MV); (6) language: published in English. The RCTs were excluded if only the abstract was published or the full text was unavailable.

### Data synthesis

The primary outcome was 28-day all-cause mortality. To investigate the effect of VC on septic patients, the data of 28-day hospital mortality were pooled. The ICU-LOS, vasopressor use, duration of MV, new-onset or worsening of AKI, and change in SOFA score after enrolment. Furthermore, we performed the subgroup analysis in this meta-analysis; importantly, if the included articles including the septic and septic shock patients, we considered these patients of studies as septic patients.

### Statistical analysis

RevMan 5.3 (Cochrane IMS, Oxford, United Kingdom) and STATA 14.0 (College Station, Texas 77845 USA) were used for the random effects model analysis. The dichotomous and continuous data were assessed using the pooled risk ratio (RR) and mean difference (MD) with their 95% confidence interval (CI), respectively. The potential publication bias was assessed using the Egger's linear regression, and funnel plots were used for visually assessing asymmetry. *P* < 0.1 in Egger's test suggested low heterogeneity.

## Results

### Study selection

**Figure 1:**
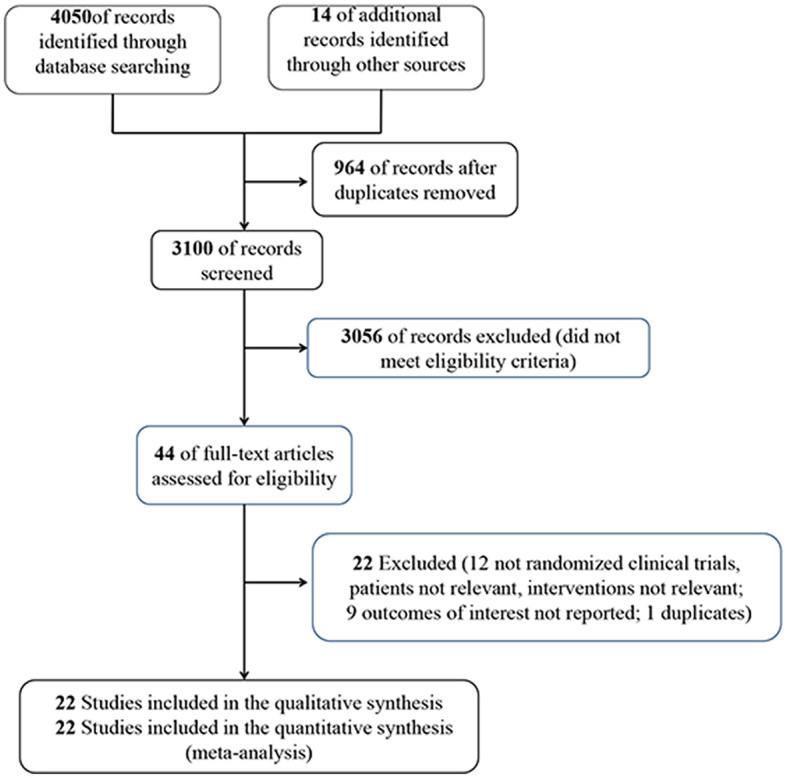
Study search strategy.

In the meta-analysis, we initially collected 4,050 references, and 3,100 remained after removing duplicates. Screening the title and abstract, and 44 RCTs were identified, of which 22 RCTs (3,570 patients) were included ultimately (Figure 1). The characteristics of the eligible studies are listed in Table 1. Ten RCTs (16, 30, 32–39) focused on 1,362 septic shock patients, while 12 RCTs (14, 15, 17, 31, 40–47) examined 2,208 septic patients. In nine RCTs (14, 16, 17, 30, 31, 36, 41, 42, 47), 1,498 patients received VC monotherapy, and in 13 RCTs (15, 32–35, 37–40, 43–46), and 2,072 patients received combination therapy.

**Table 1:** Characteristic of the included studies in the meta-analysis of vitamin C vs. placebo or standard supportive care in adult septic patients.

**References**	**Study type**	**Single/ multi- center**	**Study period**	**Total patients/ patients in VC No**.	**Mean age, y**	**Female/ male of patient No**.	**Monotherapy/ combined therapy**	**Experimental intervention**	**Reported outcomes**
Fowler et al. (14)	RCT	SC	05/2010–09/2012	24/16	NA	11/13	M	VC (50 or 200 mg/kg/d) intravenous infusion every 6 h for 4 days	28-day mortality; Days on vasopressor; Length of ICU stay; Ventilator-free days
Zabet et al. (16)	RCT	SC	09/2014–01/2016	28/14	VC: 64.14 PC: 63.71	7/21	M	VC (100 mg/kg/d) intravenous infusion every 6 h for 3 days	28-day mortality; Duration of norepinephrine administration; Length of ICU stay
Balakrishnan et al. (40)	RCT	SC	NA	24/12	VC: 55.41 PC: 53.41	9/15	C	VC (6 g/d) intravenous infusion every 6 h for 4 days, hydrocortisone (200 mg/d) intravenous infusion every 6 h for 4 days, thiamine (400 mg/d) intravenous infusion every 12 h for 4 days	Difference of SOFA score day 1/4
Fowler et al. (17)	RCT	MC	09/2014–11/2017, 01/2018	167/84	VC: 53.3 PC: 57	77/90	M	VC (200 mg/kg/d) intravenous infusion every 6 h for 4 days	28-day all-cause mortality; Modified SOFA score after 96 h; Ventilator-free days to day 28; ICU-free days to day 28
Chang et al. (15)	RCT	SC	09/2017–02/2019	80/40	VC: 59.5 PC: 63.7	37/43	C	VC (6 g/d) intravenous infusion every 6 h for 4 days hydrocortisone (200 mg/d) intravenous infusion every 6 h for 7 days and thiamine (400 mg/d) intravenous infusion every 12 h for 4 days	28-day all-cause mortality; Duration of vasopressor use, Length of ICU stay; Change in SOFA (ΔSOFA) within 72 h; New AKI after entering ICU; Duration of mechanical ventilation
Fujii et al. (35)	RCT	MC	05/2018–07/2019, 10/2019	211/107	VC: 61.9 PC: 61.6	78/133	C	VC (6 g/d) intravenous infusion every 6 h, hydrocortisone (200 mg/d) intravenous infusion every 6 h, and thiamine (400 mg/d) every 12 h intravenous infusion until shock resolution or up to 10 days	28-day mortality; up to day 7 time alive and free of vasopressors; 28-day cumulative mechanical ventilation-free days; Change in SOFA score at day 3; 28-d ICU-free days
Hwang et al. (33)	RCT	MC	12/2018–01/2020, 04/2020	111/53	VC: 69.3 PC: 68.3	69/42	C	VC (100 mg/kg/d, maximum single dose 3 g, daily dose 6 g) intravenous infusion every 12 h, thiamine (400 mg/d) intravenous infusion every 12 h for a total of 2 days	Change in SOFA score after 72 h; 28-day mortality; Vasopressor-free days; Ventilator-free days, New-onset or worsening AKI after enrolment, ICU length of stay
Iglesia et al. (44)	RCT	MC	02/2018–06/2019	137/68	VC: 70 PC: 67	78/59	C	VC (6 g/d) intravenous infusion every 6 h for 4 days, hydrocortisone (200 mg/d) intravenous infusion every 6 h for 4 days, thiamine (400 mg/d) intravenous infusion every 12 h for a maximum of 4 days	Hospital mortality; Change in SOFA score after 72 h; Duration of vasopressors; Length of ICU stay; Ventilator-free days; AKI
Mohamed et al. (38)	RCT	SC	06/2018–08/2019	88/45	VC: 59.37 PC: 58.69	25/63	C	VC (6 g/d) intravenous infusion every 6 h for 4 days, hydrocortisone (200 mg/d) intravenous infusion every 6 h for 4 days, and thiamine (400 mg/d) intravenous infusion every 12 h for 4 days	All-cause mortality; Change in SOFA score after 72 h; Incidence of new onset of AKI; Length of ICU stay
Moskowitz et al. (34)	RCT	MC	02/2018– 11/2019	200/101	VC: 68.9 PC: 67.7	89/111	C	VC (6 g/d) intravenous infusion every 6 h for 4 days, hydrocortisone (200 mg/d) intravenous infusion every 6 h for 4 days, and thiamine (400 mg/d) intravenous infusion every 6 h for 4 days	Change in SOFA score after 72 h; All-cause mortality over 30 days; Ventilator-free days; ICU-free days during the first 28 days after Enrolment; kidney failure
Rosengrave et al. (36)	RCT	SC	NA	40/20	VC: 69.7 PC: 64.6	40/27	M	VC (100 mg/kg/d) intravenous infusion every 6 h for 4 days	Duration of vasopressor administration; SOFA score at 96 h; ICU length of stay; 30-day mortality
Wani et al. (45)	RCT	SC	04/2018–06/2019	100/50	VC: 70 PC: 65	41/59	C	VC (6 g/d) intravenous infusion every 6 h for 4 days, hydrocortisone (200 mg/d) intravenous infusion every 6 h for 7 days, and thiamine (400 mg/d) intravenous infusion every 12 h for 4 days	30-day mortality; Duration of vasopressor use; SOFA score at day 4
Hussein et al. (37)	RCT	SC	08/2019–11/2020	94/47	VC: 61.60 PC: 65.81	43/51	C	VC (6 g/d) intravenous infusion every 6 h for 4 days, hydrocortisone (200 mg/d) intravenous infusion every 6 h for 4 days, and thiamine (400 mg/d) intravenous infusion every 12 h for 4 days	28-day in-hospital mortality; Vasopressors duration; ICU length of stay; Weaning from mechanical ventilation; Change in SOFA score after 96 h
Mahmoodpoor et al. (42)	RCT	SC	05/2019–12/2019	80/40	VC: 56.93 PC: 58.25	34/46	M	VC (60 mg/kg/day) as a continuous infusion for 4 days	Change of SOFA score after 96 h; Length of ICU stay; Duration of Vasopressor use; Duration of mechanical ventilation; 28-day mortality; AKI
Lyu et al. (32)	RCT	SC	02/2019–09/2021	408/205	VC: 69.0 PC: 70.5	135/273	C	VC (8 g/d) intravenous infusion every 6 h for 5 days, hydrocortisone (200 mg/d) intravenous infusion every day for 5 days, and thiamine (400 mg/d) every 12 h for 5 days	28-day mortality; 72-h Delta SOFA score; ICU-free days; ventilator support-free days up to day 28; Vasopressor-free days; ICU length of stay (LOS)
Sevransky et al. (43)	RCT	MC	08/2018–01/2020	501/252	VC: 60.6 PC: 61	228/273	C	VC (6 g/d) intravenous infusion every 6 h for 4 days, hydrocortisone sodium succinate (200 mg/d) intravenous infusion every 6 h for 4 days, thiamine hydrochloride (400 mg/d) intravenous infusion every 6 h for 4 days	Change in SOFA score; Length of ICU stay; 30-day mortality
Lv et al. (41)	RCT	SC	06/2017–05/2019	117/61	VC: 58.7 PC: 60.2	58/59	M	VC (6 g/d) intravenous infusion every 6 h until ICU discharge	28-day mortality; Change in SOFA score after 72 h; ICU stay; Application time of vasoactive drugs
Yadav et al. (46)	RCT	SC	07/2018–06/2019	60/30	VC: 36.7 PC: 37.5	22/38	C	VC (6 g/d) intravenous infusion every 6 h for 5 days, hydrocortisone (200 mg/d) intravenous infusion every 6 h for 5 days, thiamine (400 mg/d) intravenous infusion every 12 h for 5 days	SOFA score at day 5; Duration of ICU stay
Wacker et al. (30)	RCT	MC	01/2018–06/2020	124/60	VC: 68.9 PC: 73.0	61/63	M	VC (3 g/d) continuous infusion for 4 days	28-day all-cause mortality; Duration of ICU; Paired improvement in SOFA score; Duration of vasopressors; Duration of mechanical ventilation following initiation
Zhang et al. (47)	RCT	MC	02/2020–05/2020	56/27	VC: 66.3 PC: 67.0	20/36	M	VC (24 g/d) intravenously every 12 h for 7 days	28-day mortality; ICU stay; AKI
Jamshidi et al. (39)	RCT	SC	05/2018–11/2018	58/29	VC: 45.4 PC: 45.4	11/47	C	VC (6 g/d) intravenous infusion every 6 h for 3 days, hydrocortisone (200 mg/d) intravenous infusion every 6 h for 3 days, and thiamine (400 mg/d) intravenous infusion every 12 h for 3 days	Time to receiving vasopressor; SOFA score after 72 h
Lamontagne et al. (31)	RCT	MC	11/2018–01/2022	862/429	VC: 65.0 PC: 65.2	324/538	M	VC (200 mg/kg/d) intravenous infusion every 6 h for up to 4 days	28-day mortality; SOFA score at day 4; Stage 3 AKI; Vasopressor infusion days in survivors; Invasive mechanical ventilation days in survivors; Length of ICU stay

RCT, randomized controlled trial; MC, multi-center; SC, single-center; VC, vitamin C; PC, placebo or control; ICU, intensive care unit; NA, not acquired; SOFA, sequential organ failure assessment; M, monotherapy; C, combined therapy; AKI, acute kidney injury; IV, intravenous infusion.

### Primary outcomes

**Figure 2:**
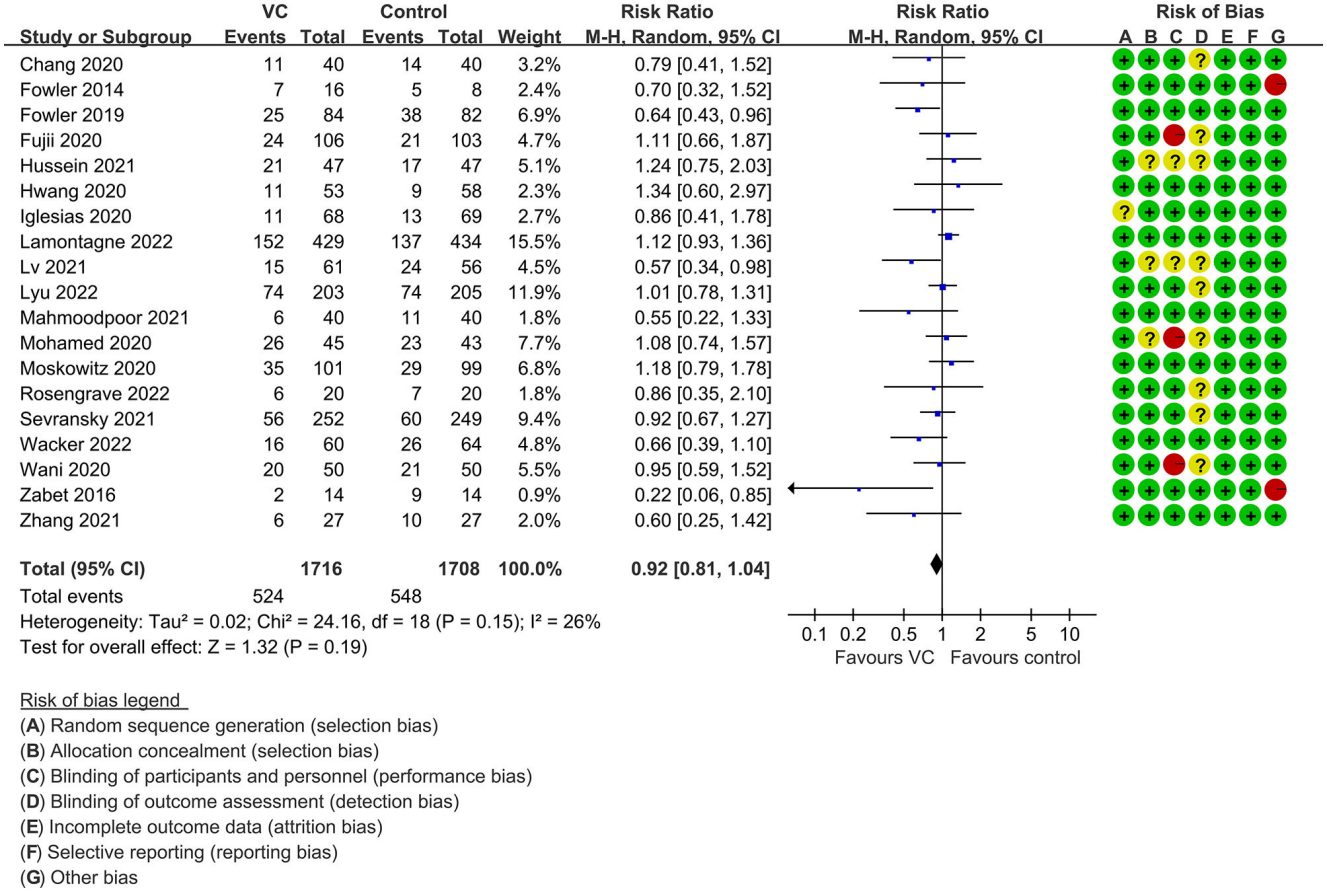
Forest plot assessing primary outcomes in septic patients based on IVVC administration.

**Figure 3:**
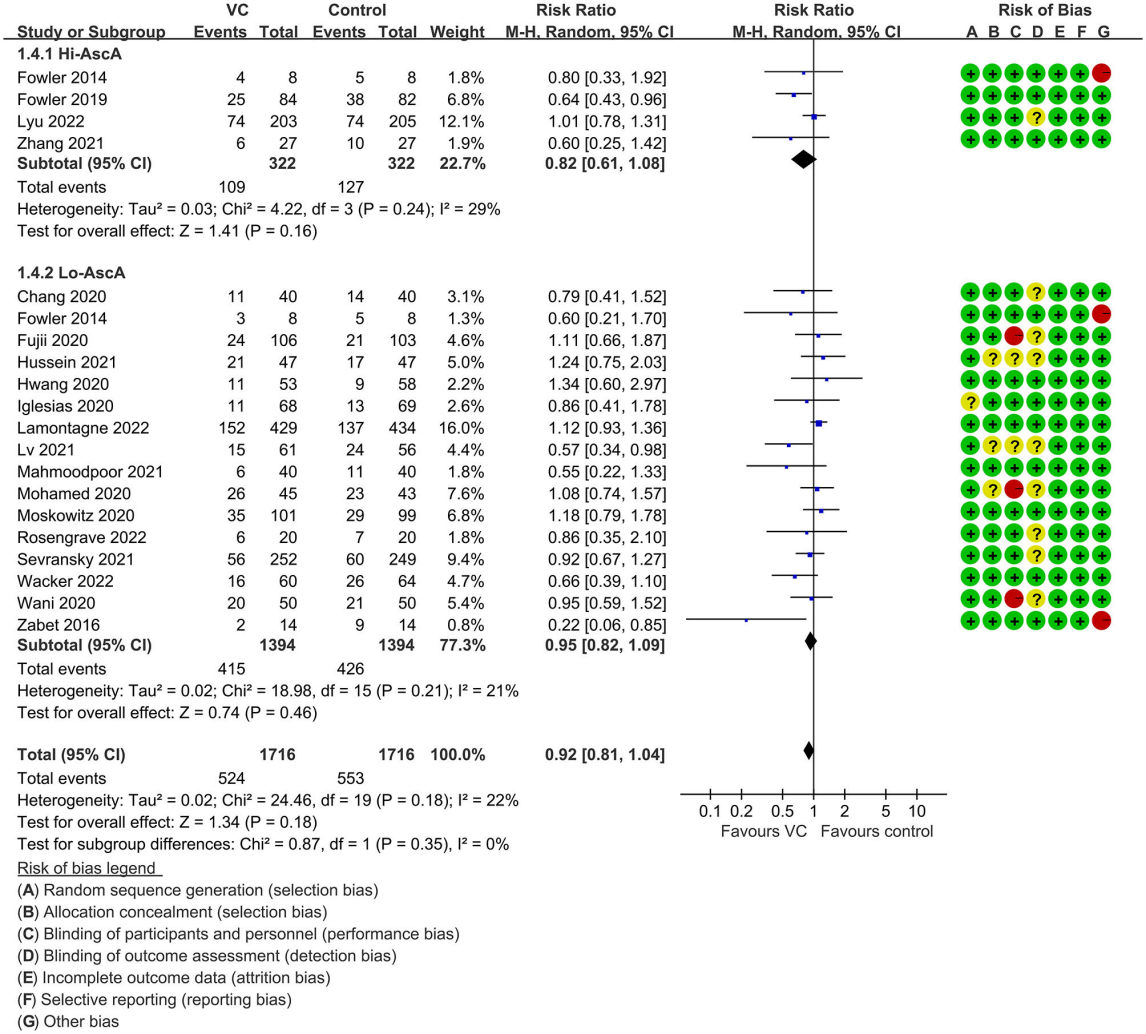
Subgroup analysis of primary outcomes in septic patients based on IVVC therapy for patients with high-dose VC (more than 100 mg/kg/d or 6 g/d) administration or low-dose administration (50–100 mg/kg/d or 6 g/d).

**Figure 4:**
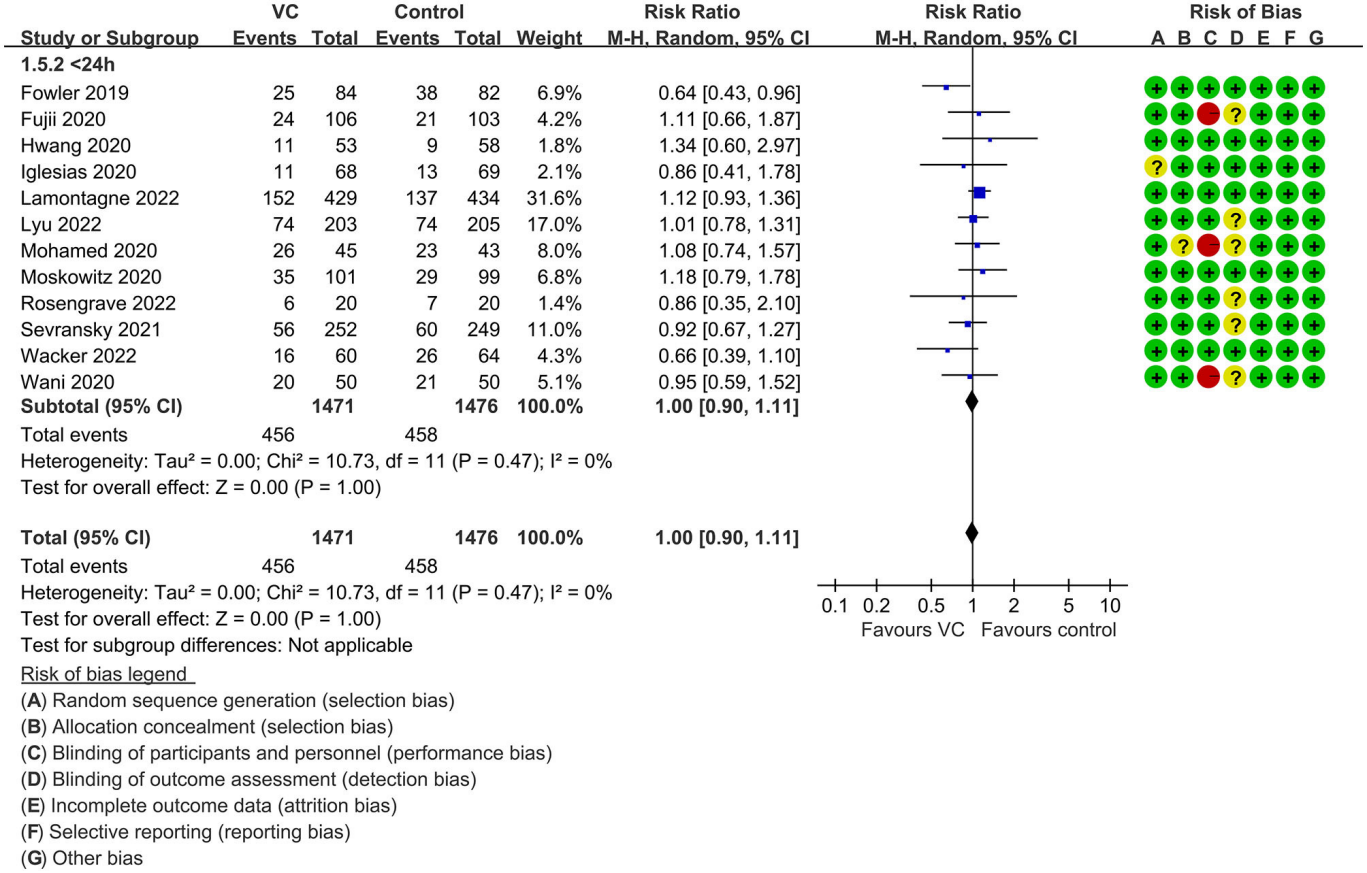
Subgroup analysis of primary outcomes in septic patients based on IVVC therapy for patients with therapy initiation within 24 h of ICU admission or sepsis diagnosis.

**Figure 5:**
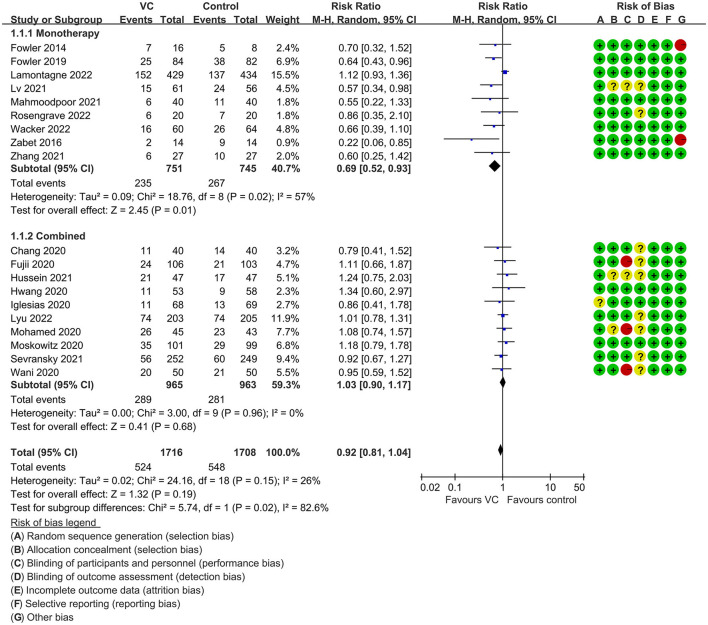
Subgroup analysis of primary outcomes in septic patients based on IVVC monotherapy or combination therapy.

**Figure 6:**
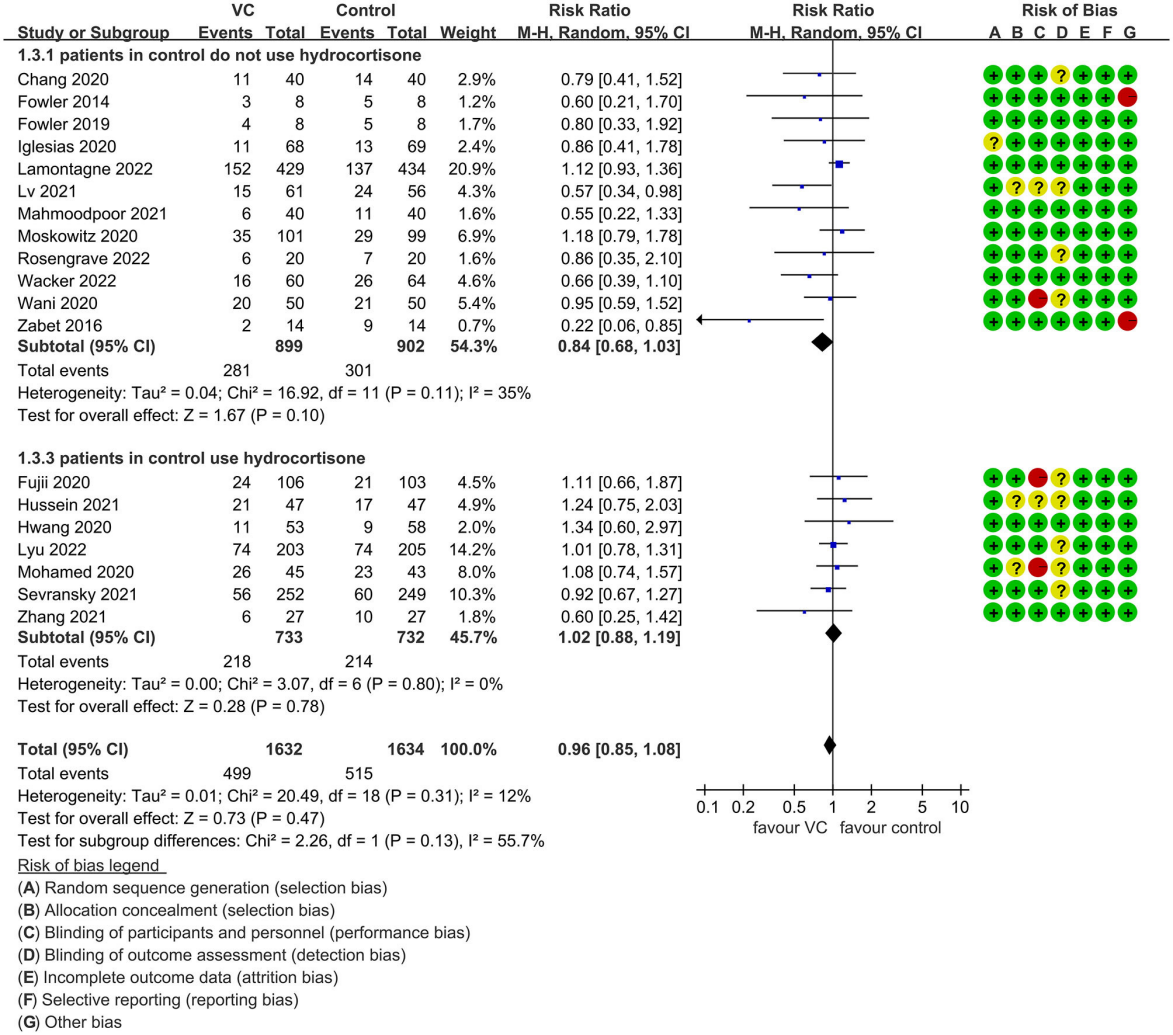
Subgroup analysis of primary outcomes in septic patients based on IVVC therapy for patients with or without hydrocortisone use in the control group.

**Figure 7:**
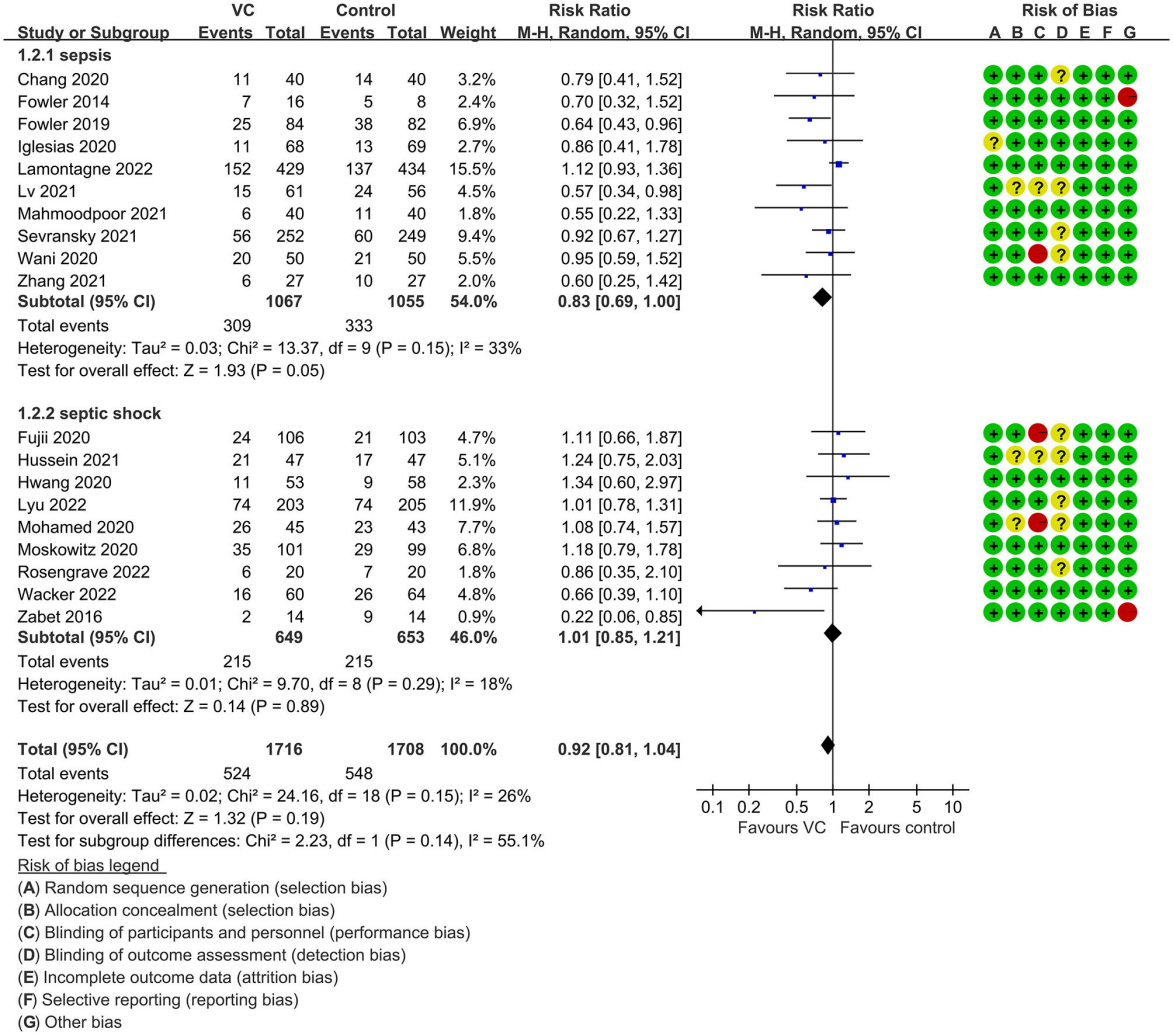
Subgroup analysis of primary outcomes in septic patients based on IVVC therapy for patients with sepsis or septic shock.

**Figure 8:**
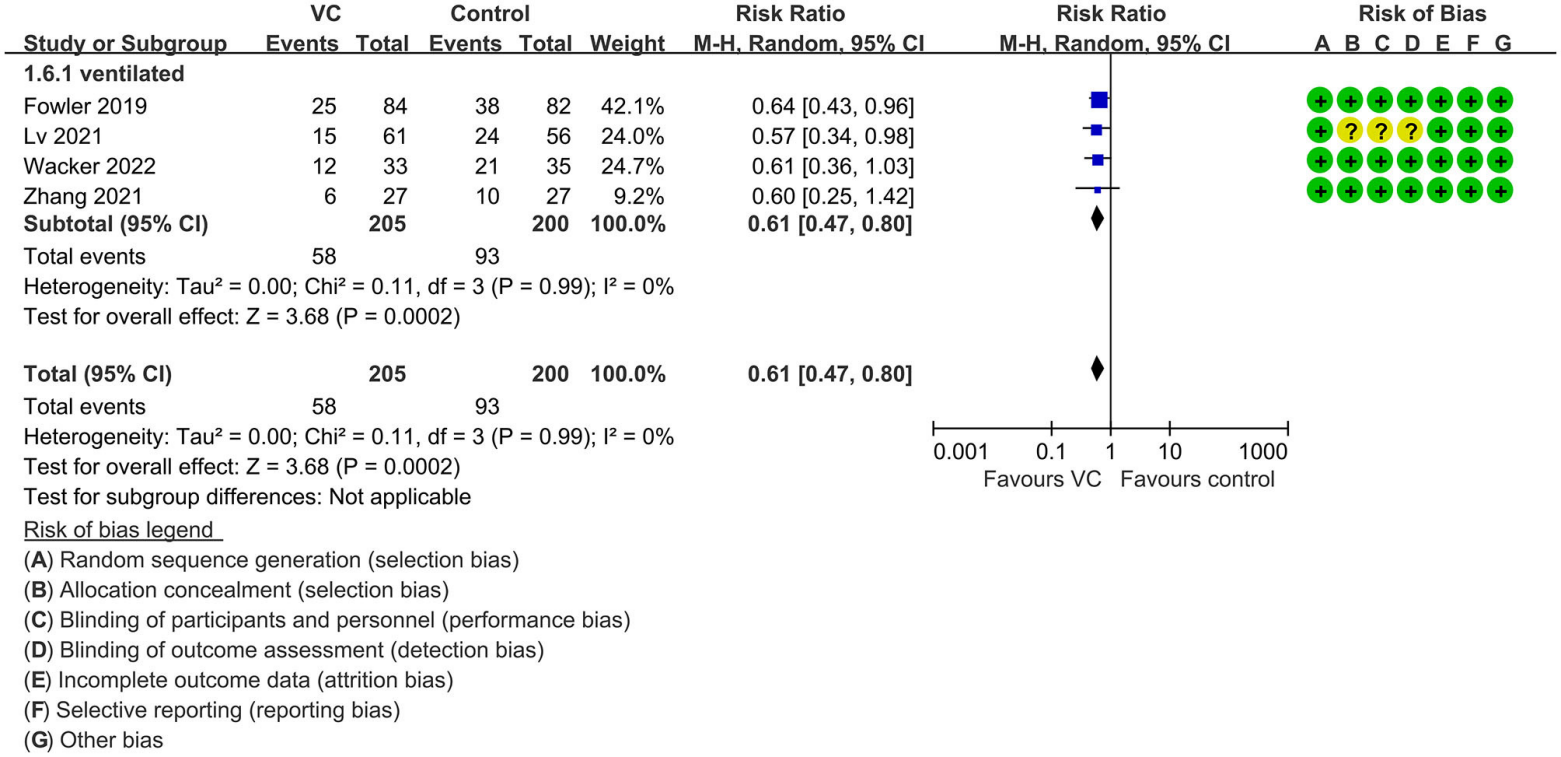
Subgroup analysis of primary outcomes in septic patients based on IVVC therapy for ventilated patients.

Our meta-analysis indicated that VC could not improve 28-day overall mortality (RR, 0.92; 95% CI, 0.81–1.04; *I*^2^ = 26%; evidence risk, moderate; Figure 2). Nine studies (14, 16, 17, 30, 31, 36, 41, 42, 47) examined VC monotherapy for adult septic patients, while 10 (15, 32–35, 37, 38, 43–45) investigated the effect of combination therapy, which mostly involved IVVC combined with intravenous thiamine and intravenous hydrocortisone. No potential publication bias was found in the primary outcome of this study (*P* = 0.559; Supplementary Figure 1). Sensitivity analysis indicated that the models were credible (Supplementary Figure 2). IVVC exhibited no effect to mortality in patients treated with a high dose of VC (>100 mg/d or >6 g/d; RR, 0.82; 95% CI, 0.61–1.08; *I*^2^ = 29%; Figure 3). Similarly, a low dose of VC had no effect on mortality (RR, 0.95; 95% CI, 0.82–1.09; *I*^2^ = 21%; Figure 3). Additionally, in 12 trials (17, 30–36, 38, 43–45), therapy was administered <24 h from ICU admission, and mortality was not affected by VC (RR, 1.00; 95% CI, 0.90–1.11; *I*^2^ = 0%; Figure 4). Subgroup analysis demonstrated that IVVC administration (monotherapy) was associated with a decrease in 28-day mortality (RR, 0.69; 95% CI, 0.52–0.93; *I*^2^ = 57%; Figure 5). However, the combination of IVVC with other medicines had no effect on all-cause mortality in septic patients (RR, 1.03; 95% CI, 0.90–1.17; *I*^2^ = 0%; Figure 5). We also analyzed the difference in mortality concerning whether hydrocortisone was used in the control group. We observed that the mortality of patients in the intervention group was not improved compared to patients without hydrocortisone therapy in the control group (RR, 0.84; 95% CI, 0.68–1.03; *I*^2^ = 35%; Figure 6). In the subgroup analysis of patients treated with hydrocortisone, mortality in the control group showed no difference compared to that in the VC group (RR, 1.02; 95% CI, 0.88–1.19; *I*^2^ = 0%; Figure 6). Additionally, 10 RCTs (14, 15, 17, 31, 41–45, 47) included septic patients, and nine trials enrolled patients with septic shock (16, 30, 32–38). IVVC treatment was associated with a trend reduction in 28-day mortality in septic patients (RR, 0.83; 95% CI, 0.69–1.00; *I*^2^ = 33%; Figure 7), whereas it did not affect septic shock patients (RR, 1.01; 95% CI, 0.85–1.21; *I*^2^ = 18%; Figure 7). For ventilated patients (17, 30, 41, 47), mortality was reduced by parenteral IVVC treatment (RR, 0.61; 95% CI, 0.47–0.80; *I*^2^ = 0%; Figure 8).

### Secondary outcomes

The IVVC treatment showed no difference in AKI incidence (RR, 1.05; 95% CI, 0.94–1.18; *I*^2^ = 0%; Supplementary Figure 3), ICU-LOS (MD, 0.07; 95% CI, −0.54–0.68; *I*^2^ = 42%; Supplementary Figure 4), change in SOFA score (MD, 0.04; 95% CI, −0.55–0.63; *I*^2^ = 96%; Supplementary Figure 5), and duration of MV (MD, 0.96; 95% CI, −0.27–2.18; *I*^2^ = 84%; evidence risk, moderate; Supplementary Figure 6). IVVC treatment for septic patients could reduce the vasopressor duration (MD, −8.45; 95% CI, −15.43 to −1.47; *I*^2^ = 80%; evidence risk, very low; Supplementary Figure 7). The funnel plot and Egger's test showed no publication bias in AKI incidence (*P* = 0.128), ICU-LOS (*P* = 0.784), change in SOFA score (*P* = 0.92), duration of MV (*P* = 0.324), and duration of vasopressor use (*P* = 0.145; Supplementary Figures 8–12) for funnel plot. This meta-analysis indicated that the models of AKI incidence, ICU-LOS, change in SOFA score, duration of MV, and duration of vasopressor use were credible (Supplementary Figures 13–17). Subgroup analysis showed that IVVC monotherapy used in seven trials (14, 16, 30, 31, 36, 41, 42) was association with lower risk of the duration of vasopressor use (MD, −12.11; 95% CI, −21.46 to −2.75, *I*^2^ = 74%; Supplementary Figure 18), but combination therapy used in eight trials (15, 32, 33, 35, 37, 39, 44, 45) exhibited no effect (MD, −4.82; 95% CI, −16.00–6.36; *I*^2^ = 83%; Supplementary Figure 18). Furthermore, IVVC administration may be related with a reduction in both duration of vasopressor use < 7 days (MD, −15.06; 95% CI, −23.43 to −6.70; *I*^2^ = 0%; Supplementary Figure 19) and duration of vasopressor use ≥ 7 days (MD, −7.06; 96% CI, −15.05–0.93; *I*^2^ = 82%; Supplementary Figure 19). Furthermore, the risk of bias and the evidence rank of each outcome in this meta-analysis are tabulated in Table 2.

**Table 2:** Findings and evidence rank of the included studies with vitamin C vs. placebo treatment in septic patients.

**VC for sepsis**						
Patient or population: septic patients						
Settings:						
Intervention: VC						
Outcomes	Illustrative comparative risks^*^ (95% CI)		Relative effect (95% CI)	No of participants (studies)	Quality of the evidence (GRADE)	Comments
	Assumed risk	Corresponding risk				
	Control	VC				
28-day mortality	Study population		RR 0.92 (0.81–1.04)	3,424 (19 studies)	⊕⊕⊕⊖ moderate^a^
	321 per 1,000	295 per 1,000 (260–334)			
	Moderate				
	361 per 1,000	332 per 1,000 (292–375)			
ICU length of stay		The mean ICU length of stay in the intervention groups was 0.07 higher (0.54 lower to 0.68 higher)		3,357 (19 studies)	⊕⊕⊕⊖ moderate^a^	
SOFA score		The mean SOFA score in the intervention groups was 0.04 higher (0.55 lower to 0.63 higher)		3,350 (19 studies)	⊕⊕⊕⊖ moderate^a^	
AKI	Study population		RR 1.05 (0.94–1.18)	1,581 (eight studies)	⊕⊕⊕⊕ high
	344 per 1,000	361 per 1,000 (324–406)			
	Moderate				
	248 per 1,000	260 per 1,000 (233–293)			
Mechanical ventilation		The mean mechanical ventilation in the intervention groups was 0.96 higher and (0.27 lower-2.18 higher)		1,977 (12 studies)	⊕⊕⊕⊖ moderate^b^	
Vasopressor duration		The mean vasopressors duration in the intervention groups was 8.45 lower (15.43–1.47 lower)		2,205 (15 studies)	⊕⊖⊖⊖ very low^a^^,^ ^b^^,^ ^c^	

* The basis for the assumed risk (e.g., the median control group risk across studies) is provided in footnotes. The corresponding risk (and its 95%CI) is based on the assumed risk in the control group and the relative effect of the intervention group (and its 95% CI).

CI, Confidence interval; RR, Risk ratio.

GRADE Working Group grades of evidence.

High quality: Further research is very unlikely to change our confidence in the estimate of effect.

Moderate quality: Further research is likely to have an important impact on our confidence in the estimate of effect and may change the estimate.

Low quality: Further research is very likely to have an important impact on our confidence in the estimate of effect and is likely to change the estimate.

Very low quality: We are very uncertain about the estimate.

a Risk of bias.

b Inconsistency.

c Imprecision.

## Discussion

The results of this meta-analysis suggest that IVVC may not improve the 28-day overall mortality in septic patients. However, the effect of the drug is influenced by various factors such as dosage, timing of therapy, drug combination, frequency of hydrocortisone use in the control group, and severity of illness (septic shock and ventilation). There is no reduction mortality with high-dose VC and low-dose VC. Similarly, the VC therapy initiation of therapy within 24h of ICU admission or sepsis diagnosis did not impact the mortality of septic patients. Moreover, the use of IVVC monotherapy, but not combination therapy, was found to decrease the 28-day overall mortality in septic patients. In subgroup analysis, the mortality was not reduced in the control group when hydrocortisone was used. IVVC treatment was associated with a trend of decreasing the mortality of septic patients compared to septic shock patients, and ventilated patients benefited more from IVVC.

Previous met-analysis study (26) only included 10 studies showed that VC does not affect in-hospital or ICU mortality of septic patients, which differs from our study findings. However, the study (26) assessed pooled data from only three retrospective studies, making the methodology quality low. Including a small number of retrospective studies decreases the precision of the conclusion. One study (48) conducted that VC treatment or combination of glucocorticoids and vitamin B1 could not reduce the long-term mortality compare with control group, whereas it not reported the short-term mortality. A recent meta-analysis (49) compared the effect of IVVC on critical ill patients with that of a placebo and found that IVVC could reduce 28-day mortality, which was similar to our study findings. However, this study (49) is limited because of not indicating that the IVVC monotherapy reduce in the duration of vasopressor use and lack of discussion about VC do not affect AKI incidence. An additional meta-analysis (50) showed VC could reduce the 28-day mortality, whereas the author did not conduct subgroup analysis on patients with different disease severity levels, such as those with sepsis and septic shock.

Oxidant molecules play a critical role in host defense by impacting cellular signaling, regulating vascular tone, and modulating production of prostaglandins. A massive early production of oxidants followed by a quick decline may result in mitochondrial and endothelial dysfunction, which can lead to the condition deterioration in sepsis (9). Based on the problematic role of oxidants, IVVC is suggested as an aggressive early therapy, followed by quick cessation after the imbalance of ROS has been corrected. The huge burst of ROS is generated within minutes after the start of reperfusion. Therefore, a late start of vitamin infusion may lead to negative results (51). However, the subgroup analysis of early drug administration (time of therapy from ICU admission or diagnosis of sepsis or septic shock within 24 h) did not significantly influence mortality, which is contrary to our expectations. Further trials are needed to explore this finding.

Our meta-analysis suggests that IVVC monotherapy could significantly decrease the 28-day mortality, while combined therapy has no effect. However, the mechanisms underlying the beneficial effects of VC on sepsis mortality are not yet fully understood. An *in vitro* study has shown that hydrocortisone may reduce the inflammatory response by increasing the level of sodium-coupled VC transporter 2, which facilitates the uptake of VC into the cell (52). However, the recent meta-analysis showed that combining VC with other therapies did not lead to a reduction in mortality in septic patients (23). In the early stage of sepsis, the release of many cytokines and the dysregulation of the inflammatory response caused by damaged tissues can damage the vascular endothelial cells, leading to acute organ dysfunction. Therefore, the restoration of vascular endothelial integrity and capillary function, as well as the early reduction of inflammation in sepsis are important goals for treating sepsis. Based on the pharmacological mechanisms of hydrocortisone, VC, and thiamine, and the results of Chang et al.'s study, it is speculated that early combination therapy may be beneficial, but the efficacy may vary among patients with different stages of sepsis (15). VC may be beneficial during initial anabatic inflammatory responses, however at reactive immunosuppression VC is harmful to the body (53, 54). The hydrocortisone may aggravate the disadvantage of VC due to its promoting to immunosuppression. Antagonism between hydrocortisone and VC may weigher than synergism. The use of hydrocortisone for treating septic shock has been controversial, with studies yielding mixed results. Two recent RCTs, ADRENAL and APROCCHSS, suggest that glucocorticoids are effective in treating critically ill patients with septic shock (55, 56). The therapeutic effect of hydrocortisone on sepsis may be related to disease severity and medication dosage. One study demonstrated that thiamine supplementation could lower blood lactate levels in some sepsis patients with thiamine deficiency at enrollment, indicating that baseline thiamine levels may be associated with the efficacy of the Hydrocortisone, Ascorbic acid, Thiamine (HAT) regimen (57). VC may not have a synergistic effect with hormones and thiamine, and its therapeutic effect may be maximized when administered as monotherapy. Therefore, monitoring plasma levels of VC and thiamine upon admission is necessary to evaluate efficacy and guide drug administration.

The finding suggests that adding hydrocortisone and thiamine to VC therapy does not reduce 28-day mortality. VC combination therapy reportedly does not affect sepsis patients, indicating that future studies should focus on confirming the impact of VC monotherapy. Furthermore, this study showed no difference in secondary outcomes such as AKI incidence, change in SOFA score, ICU-LOS, and duration of MV between monotherapy and combination therapy. VC monotherapy is associated with a significant reduction in the duration of vasopressor use. The incidence of adverse events was extremely low, and we acknowledge that there are accidental factors and individual differences. Only one trial reported increased rates of hypernatremia following VC treatment (34). Although a patient with hypoglycemia who received VC experienced a severe episode (31), this was an iatrogenic injury unrelated to VC therapy. Moreover, three patients exhibited hypotension and tachycardia (42). Furthermore, we compared mortality between arms in subgroups where hydrocortisone monotherapy was mandated or could be used in the control group. When steroids were only used as part of the co-intervention in patients requiring high-dose vasopressors, the outcomes suggest no difference in mortality between VC and control groups.

Patients with septic shock represent the worsening subtype of sepsis. Lyu et al.'s study showed that baseline SOFA scores (mean 10) were higher in patients with septic shock. Higher SOFA scores are associated with an increased risk of death, which may be one reason why no beneficial effect of VC on mortality was observed in the study (32). VC prevents the accumulation of activated neutrophils in alveolar spaces, increasing alveolar fluid clearance in ventilated patients. This also depends on the effect of VC promoting alveolar epithelial water-channel expression and preventing damage (11). Additionally, VC reduces vascular injury by preventing neutrophil extracellular trap formation (58), suggesting a heightened mortality benefit in patients requiring ventilation.

Oxidative stress resulting from sepsis contributes to multiple organ failure. Inadequate antioxidants to counter elevated levels of ROS and nitrogen lead to cellular injury and endothelial barrier dysfunction (59, 60). VC can reduce nitric oxide production via the iNOS pathway and decrease vasoconstriction and vascular permeability (61). Additionally, plasma VC levels decrease rapidly under acute inflammation, accompanied by significant human tissue alterations, including dysregulated inflammation, increased endothelial permeability, and edema (10, 62, 63). VC is associated with increased superoxide dismutase activity and decreased malondialdehyde levels (64). With short longevity and water-solution, VC displays saturable enteral absorption kinetics, indicating that VC supplementation may improve clinical outcomes in septic patients. However, (62) it showed that suggesting the wide heterogeneity an antioxidant decreases in response to ICU patients when planning to use antioxidant therapy. Similarly, the previous article showed that (65) the antioxidant needs for personalized approaches: Right species, Right place, Right time, Right level, and Right target, suggesting precision redox is the key for antioxidant pharmacology. Furthermore, one report (66) suggested that Gut microbiota participates in the pathogenesis of sepsis by influencing the inflammatory state and immune response of the host. However, Whether VC will also affect the prognosis of sepsis patients by changing the host's gut microbiota, and it will be further to research in the future studies.

Our meta-analysis has several strengths. We searched for the latest and most comprehensive studies to estimate the effect of VC on septic patients. Importantly, we set explicit inclusion criteria to limit bias. Thirdly, all included studies were RCTs, which enhances the quality of our findings. The risk of bias was low among the eligible studies. We explored the influence of dosage, timing of therapy, drug combination, hydrocortisone frequency in the control group, and severity (septic shock and ventilation) on intravenous VC's therapeutic effect.

Some limitations of this study are noted, including that most included studies did not report baseline plasma VC levels (11, 12, 14, 54), leading to a missed signal in patients with baseline deficiency. The endpoint of secondary outcomes is various effecting the reliability. Besides, some of the sample size of subgroup is too small to get precise results and most tests of subgroup differences are not statistically significant.

## Conclusions

IVVC administration could not improve the 28-day all-cause mortality of septic patients. IVVC monotherapy, rather than combination therapy, could decrease all-cause mortality in septic patients. The findings suggest that the effect of HAT combination therapy on septic patients is not superior to IVVC monotherapy. However, subgroup analysis suggested that IVVC had a trend to reduce the 28-day mortality in patients with sepsis but not septic shock. This effect should be further confirmed in future studies.

## Data availability statement

The original contributions presented in the study are included in the article/Supplementary material, further inquiries can be directed to the corresponding author.

## Author contributions

TS and HL provided the idea for the meta-analysis. QM, SQ, LL, and ZX contributed to the data extraction. QM and LL computed the pooled outcomes. HL and QM wrote the article. TS revised the article. All authors contributed to the article and approved the submitted version.

## Supplementary material

Supplementary Figure 1 Funnel plot assessing the potential publication bias for primary outcomes in septic patients based on IVVC administration.

Supplementary Figure 2 Sensitivity analysis for assessing the robustness of primary outcomes in septic patients based on IVVC administration.

Supplementary Figure 3 Forest plot assessing the risk of AKI incidence in septic patients based on IVVC administration.

Supplementary Figure 4 Forest plot assessing the effect of ICU-LOS in septic patients based on IVVC administration.

Supplementary Figure 5 Forest plot assessing the effect of SOFA score in septic patients based on IVVC administration.

Supplementary Figure 6 Forest plot assessing the effect of duration of MV in septic patients based on IVVC administration.

Supplementary Figure 7 Forest plot assessing the effect of duration of vasopressor use in septic patients based on IVVC administration.

Supplementary Figure 8 Funnel plot assessing the potential publication bias for the effect of AKI incidence in septic patients.

Supplementary Figure 9 Funnel plot assessing the potential publication bias for the effect of ICU-LOS in septic patients.

Supplementary Figure 10 Funnel plot assessing the potential publication bias for the effect in SOFA score in septic patients.

Supplementary Figure 11 Funnel plot assessing the potential publication bias for the duration of MV in septic patients.

Supplementary Figure 12 Funnel plot assessing the potential publication bias for the duration of vasopressor use in septic patients.

Supplementary Figure 13 Sensitivity analysis assessing the robustness of AKI incidence in septic patients based on IVVC administration.

Supplementary Figure 14 Sensitivity analysis assessing the robustness of ICU-LOS in septic patients based on IVVC administration.

Supplementary Figure 15 Sensitivity analysis assessing the robustness of SOFA score of septic patients based on IVVC administration.

Supplementary Figure 16 Sensitivity analysis for assessing the robustness of the duration of MV in septic patients based on IVVC administration.

Supplementary Figure 17 Sensitivity analysis assessing the robustness of the duration of vasopressor use in septic patients based on IVVC administration.

Supplementary Figure 18 Subgroup analysis of the duration of vasopressor use in septic patients based on IVVC monotherapy or combination therapy.

Supplementary Figure 19 Subgroup analysis of the duration of vasopressor use in septic patients based on IVVC therapy.

Supplementary Table 1 Detailed search strategy.
