# Supplementary material for: Effect of intravenous vitamin C on adult septic patients: a systematic review and meta-analysis
Source: Front Nutr. 2023 Aug 3;10:1211194. doi: 10.3389/fnut.2023.1211194 (PMC10437115; doi:10.3389/fnut.2023.1211194)
Supplement: Supplementary Figure 1 — Funnel plot assessing the potential publication bias for primary outcomes in septic patients based on IVVC administration. [file Data_Sheet_1.zip › Supplemental Figures/STable 1.DOCX]

| **Electronic databases** | **Search** | **Search strategy** | **Results** |
| --- | --- | --- | --- |
| **MEDLINE** | #1 | ("sepsis"[Title/Abstract] OR "sepsis"[MeSH Terms] OR (("critical illness"[MeSH Terms] OR ("critical"[All Fields] AND "illness"[All Fields]) OR "critical illness"[All Fields] OR ("critically"[All Fields] AND "ill"[All Fields]) OR "critically ill"[All Fields]) AND "patients"[MeSH Terms]) OR "critically ill patients"[Title/Abstract]) | 235,278 |
|  | #2 | "COVID-19"[Title/Abstract] OR "COVID-19"[MeSH Terms] | 301,162 |
|  | #3 | ("vitamin c"[Title/Abstract] OR "ascorbic acid"[Title/Abstract] OR "ascorbic acid"[MeSH Terms] OR "ascorbic acid"[MeSH Terms]) | 71,693 |
|  | #4 | #1 OR #2 | 531,672 |
|  | #5 | #3 AND #4 | **950** |
| **EMBASE** | #1 | sepsis:ti,ab,kw OR 'critically ill patients':ti,ab,kw OR 'sepsis'/de OR 'critically ill patients' | 410,613 |
|  | #2 | 'ascorbic acid':ti,ab,kw OR 'ascorbic acid'/de | 119,908 |
|  | #3 | 'coronavirus disease 2019'/exp OR 'coronavirus disease 2019' | 281,937 |
|  | #4 | #1 OR #3 | 682,932 |
|  | #5 | #2 AND #4 | **2,764** |
| **Cochrane CENTRAL** | #1 | ("ascorbic acid" or "vitamin C"):ti,ab,kw | 6,088 |
|  | #2 | MeSH descriptor: [Ascorbic Acid] this term only | 2,390 |
|  | #3 | #1 OR #2 | 6,088 |
|  | #4 | ("sepsis" or "critically ill patients"):ti,ab,kw | 17,364 |
|  | #5 | MeSH descriptor: [Sepsis] this term only | 2,329 |
|  | #6 | (COVID-19):ti,ab,kw | 12,761 |
|  | #7 | MeSH descriptor: [COVID-19] this term only | 2,447 |
|  | #8 | #4 or #5 or #6 #7 | 29,763 |
|  | #9 | #3 AND #6 | **336** |

**STable 1. The detailed search strategy**
