# Supplementary figures and images for: Effect of intravenous vitamin C on adult septic patients: a systematic review and meta-analysis
Source: Front Nutr. 2023 Aug 3;10:1211194. doi: 10.3389/fnut.2023.1211194 (PMC10437115; doi:10.3389/fnut.2023.1211194)

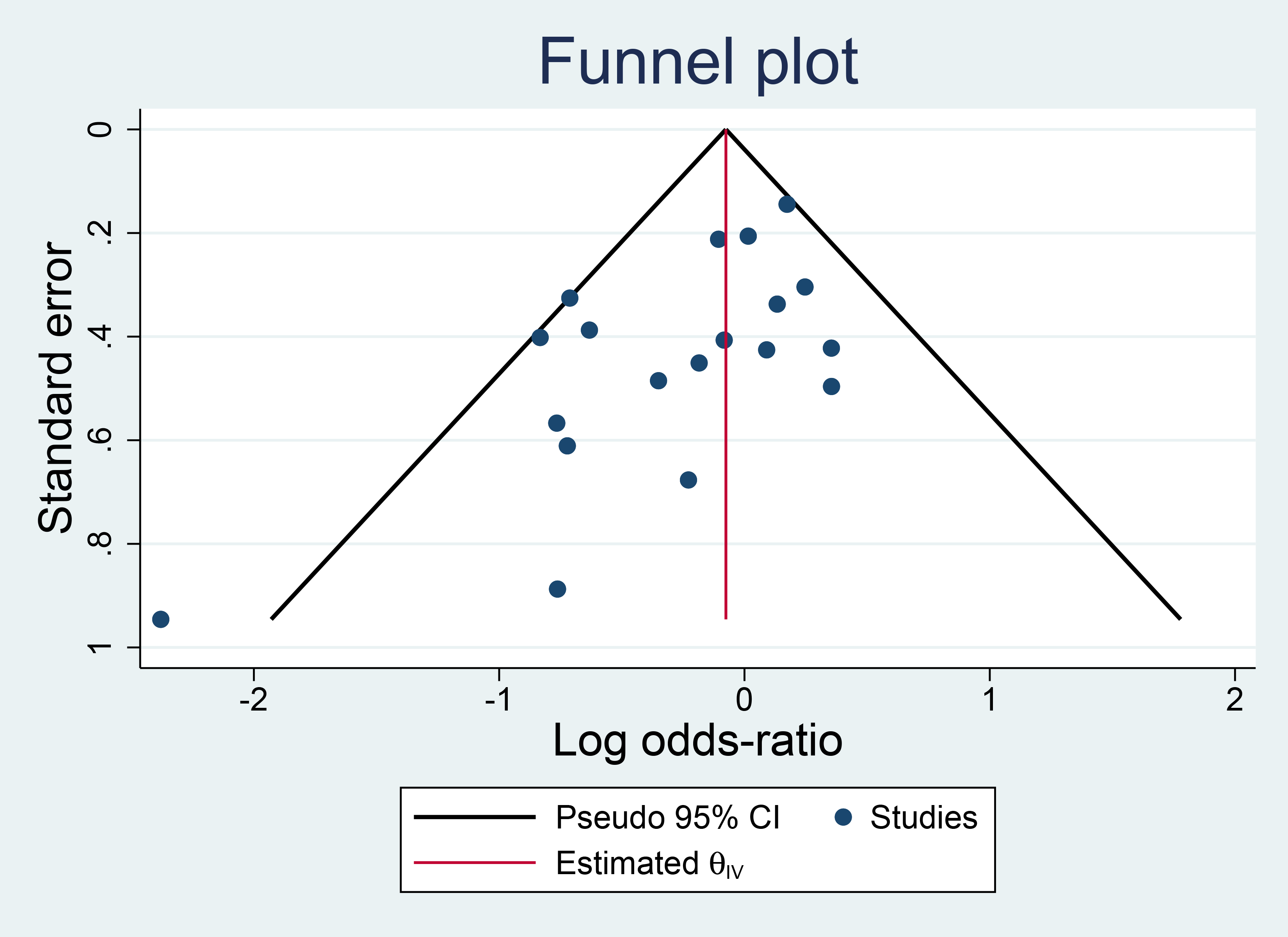

Supplement: Supplementary Figure 1 — Funnel plot assessing the potential publication bias for primary outcomes in septic patients based on IVVC administration. [file Data_Sheet_1.zip › Supplemental Figures/SFig 1.TIF]

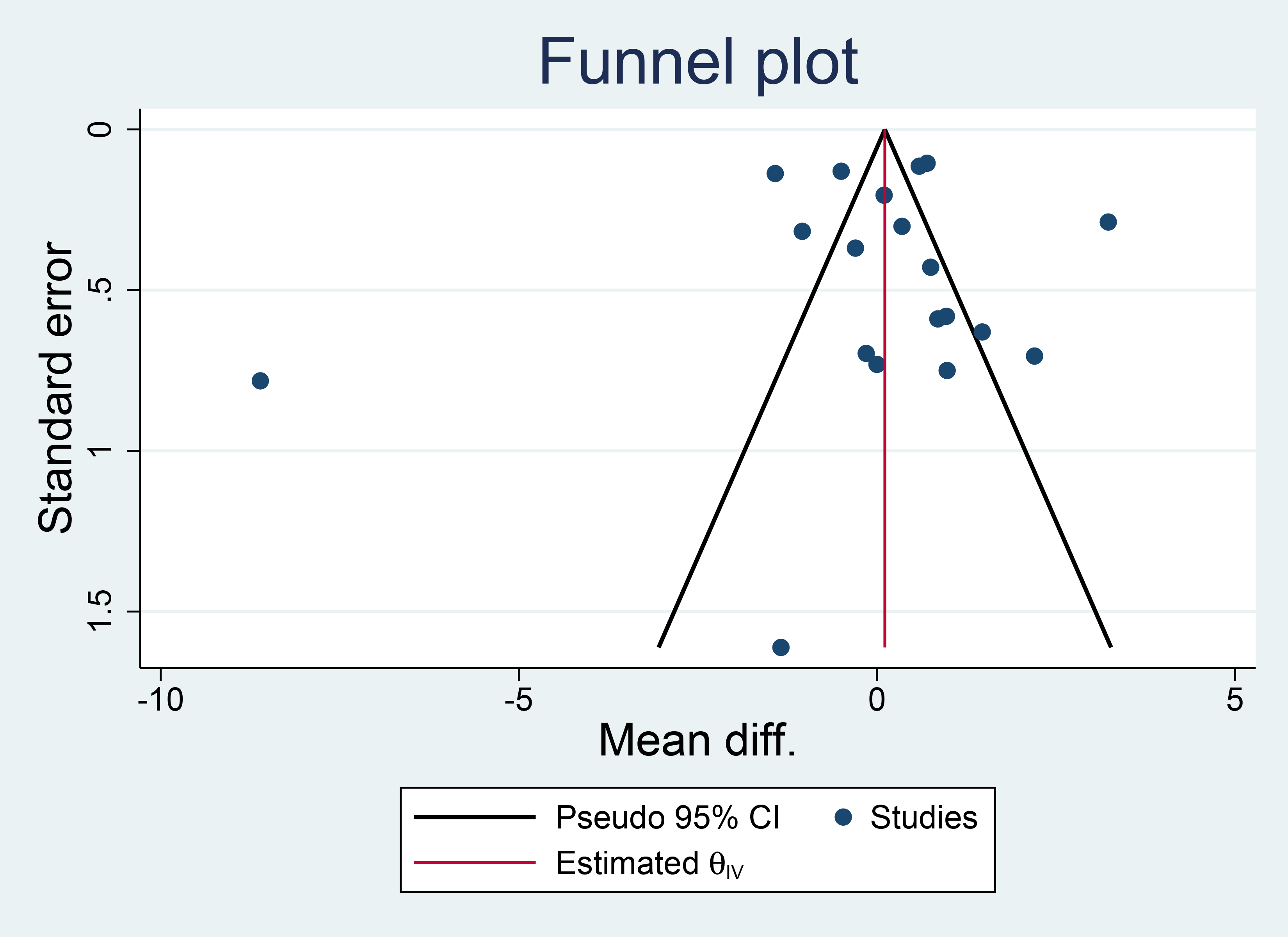

Supplement: Supplementary Figure 1 — Funnel plot assessing the potential publication bias for primary outcomes in septic patients based on IVVC administration. [file Data_Sheet_1.zip › Supplemental Figures/SFig 10.TIF]

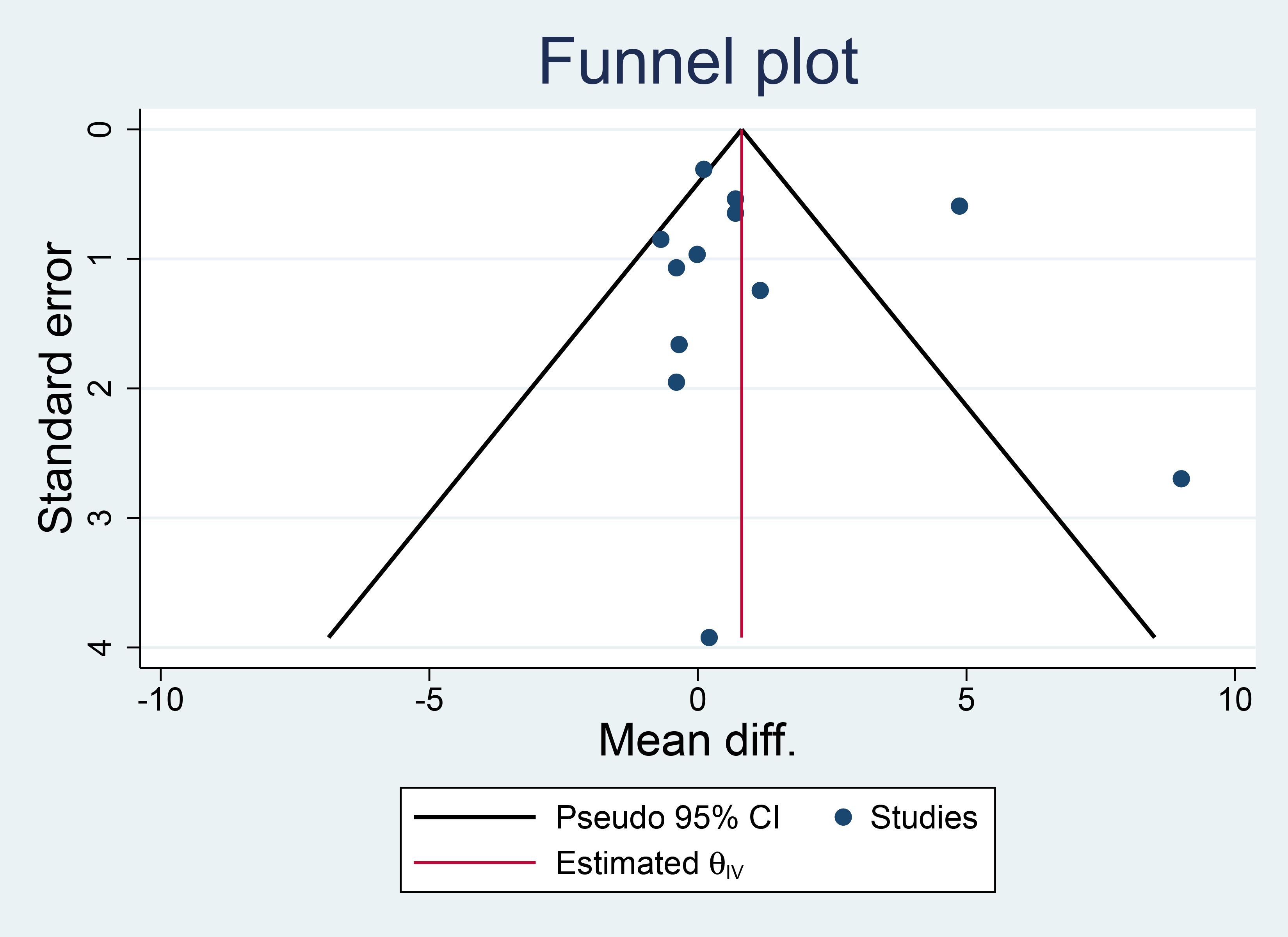

Supplement: Supplementary Figure 1 — Funnel plot assessing the potential publication bias for primary outcomes in septic patients based on IVVC administration. [file Data_Sheet_1.zip › Supplemental Figures/SFig 11.TIF]

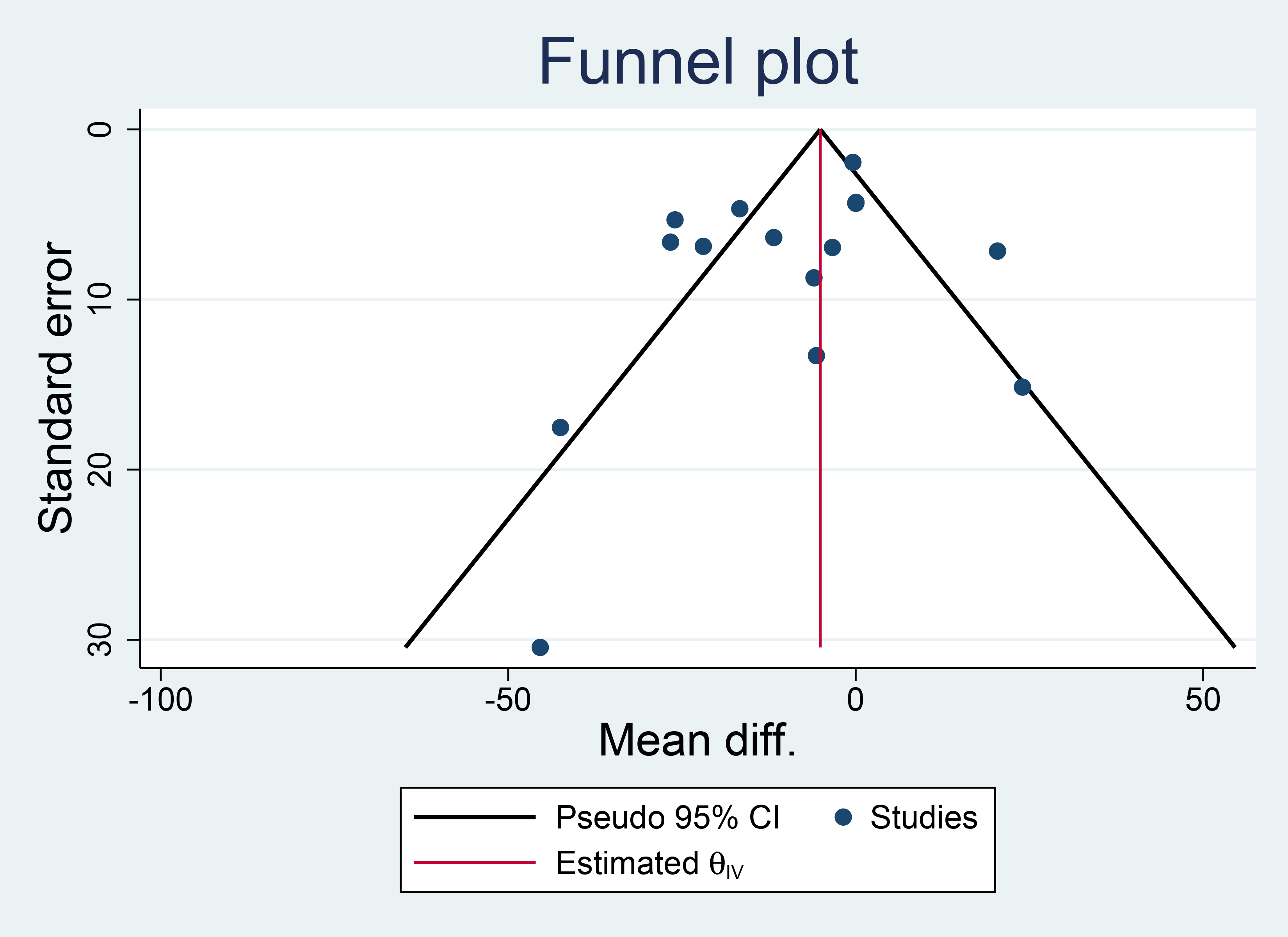

Supplement: Supplementary Figure 1 — Funnel plot assessing the potential publication bias for primary outcomes in septic patients based on IVVC administration. [file Data_Sheet_1.zip › Supplemental Figures/SFig 12.TIF]

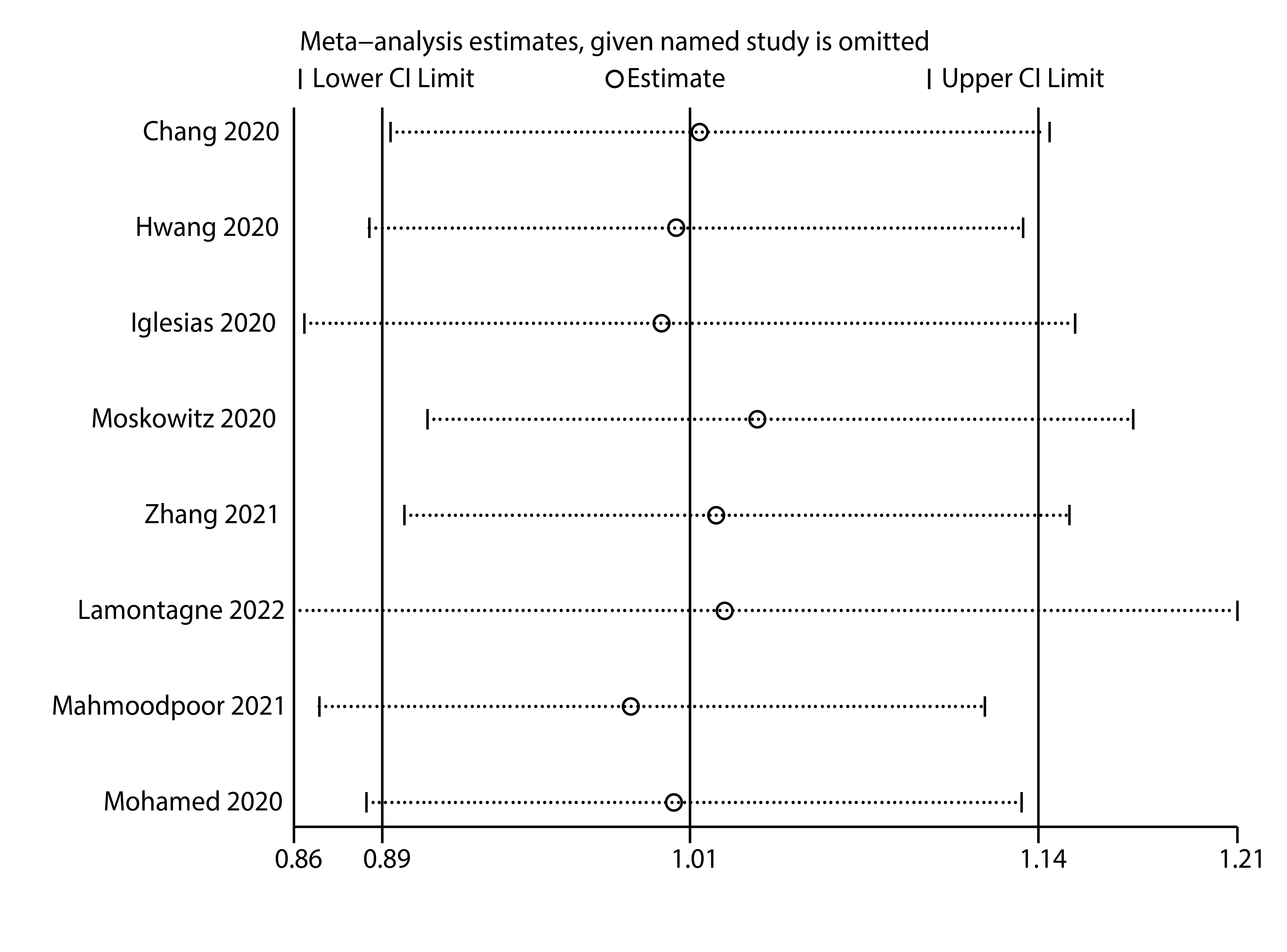

Supplement: Supplementary Figure 1 — Funnel plot assessing the potential publication bias for primary outcomes in septic patients based on IVVC administration. [file Data_Sheet_1.zip › Supplemental Figures/SFig 13.TIF]

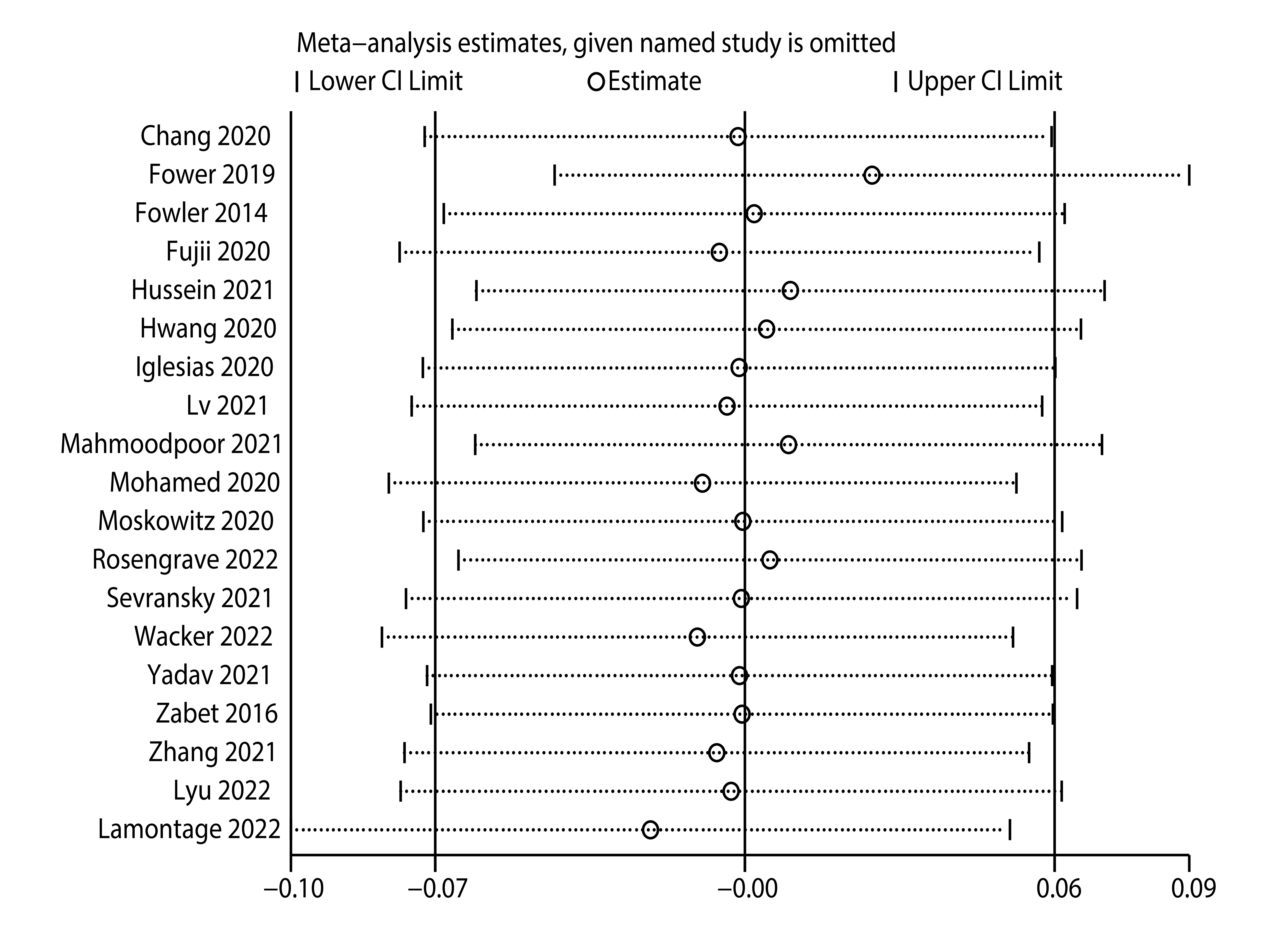

Supplement: Supplementary Figure 1 — Funnel plot assessing the potential publication bias for primary outcomes in septic patients based on IVVC administration. [file Data_Sheet_1.zip › Supplemental Figures/SFig 14.TIF]

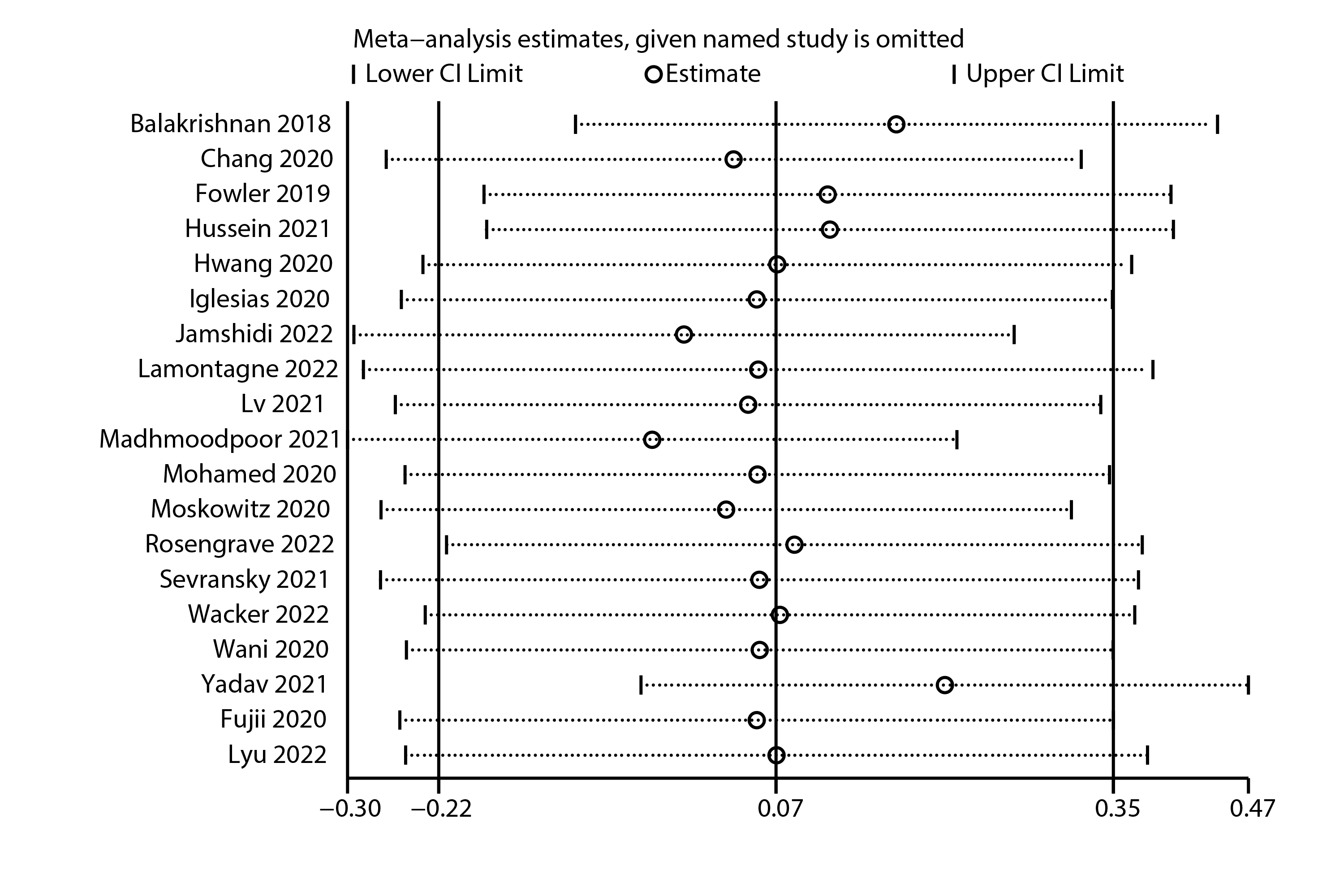

Supplement: Supplementary Figure 1 — Funnel plot assessing the potential publication bias for primary outcomes in septic patients based on IVVC administration. [file Data_Sheet_1.zip › Supplemental Figures/SFig 15.TIF]

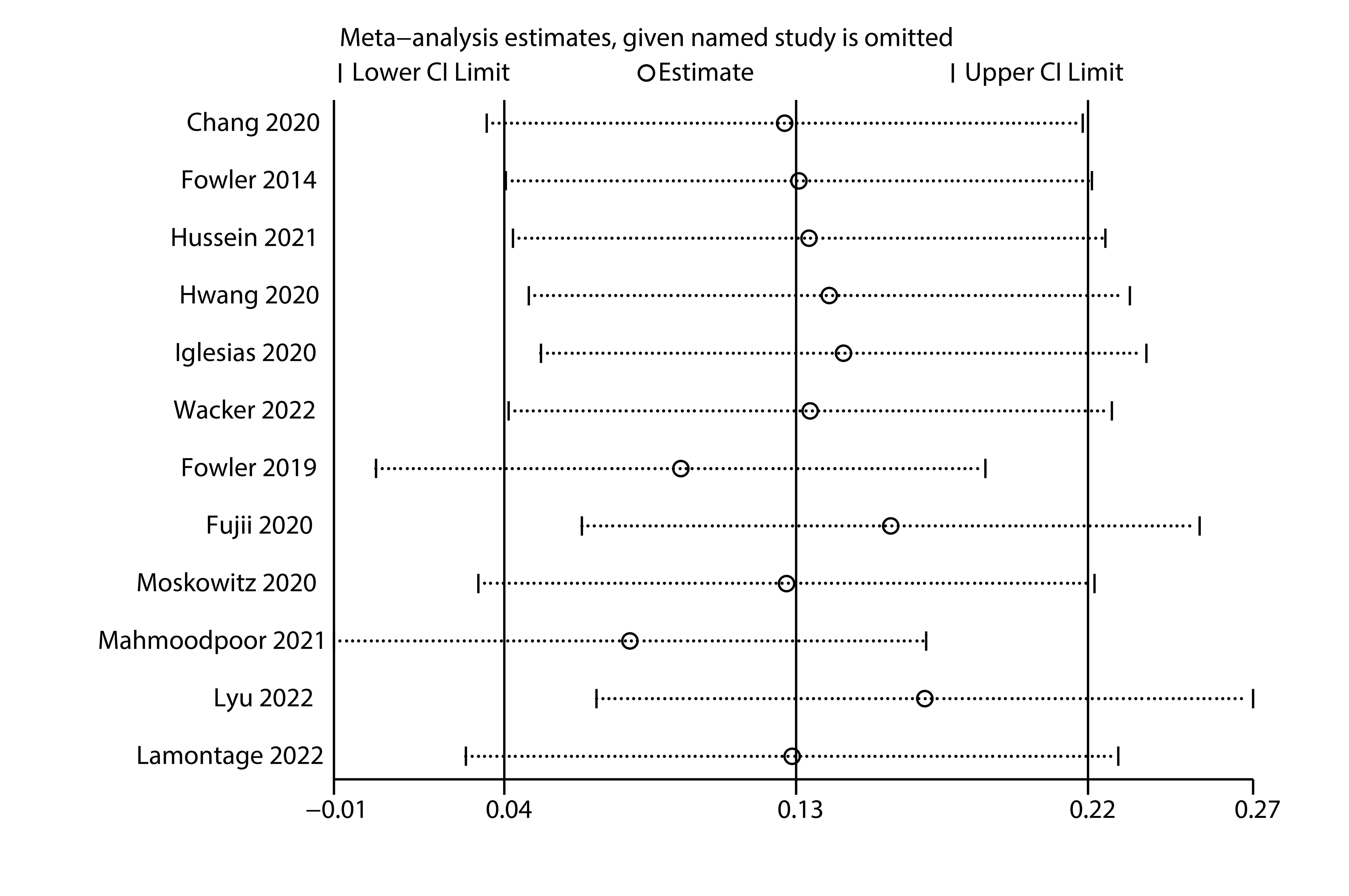

Supplement: Supplementary Figure 1 — Funnel plot assessing the potential publication bias for primary outcomes in septic patients based on IVVC administration. [file Data_Sheet_1.zip › Supplemental Figures/SFig 16.tif]

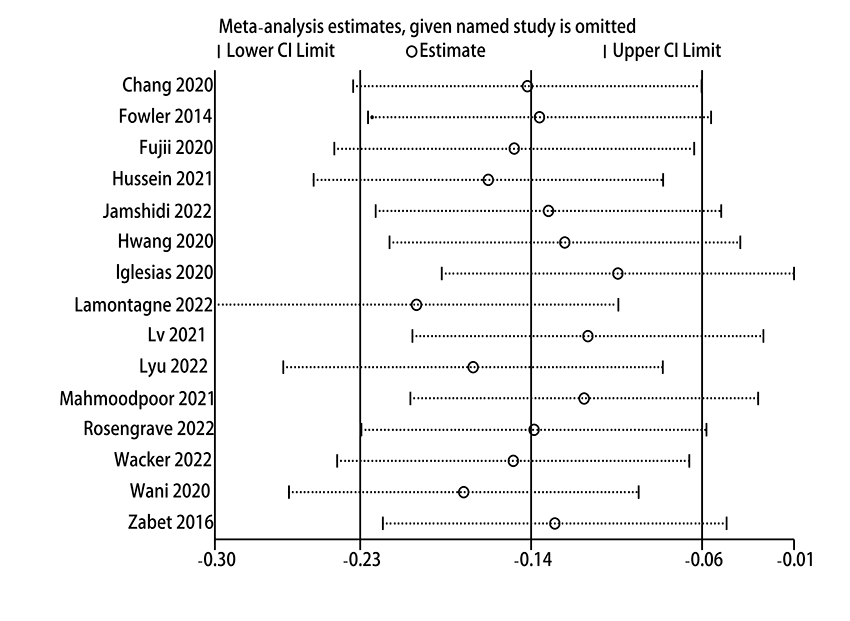

Supplement: Supplementary Figure 1 — Funnel plot assessing the potential publication bias for primary outcomes in septic patients based on IVVC administration. [file Data_Sheet_1.zip › Supplemental Figures/SFig 17.tif]

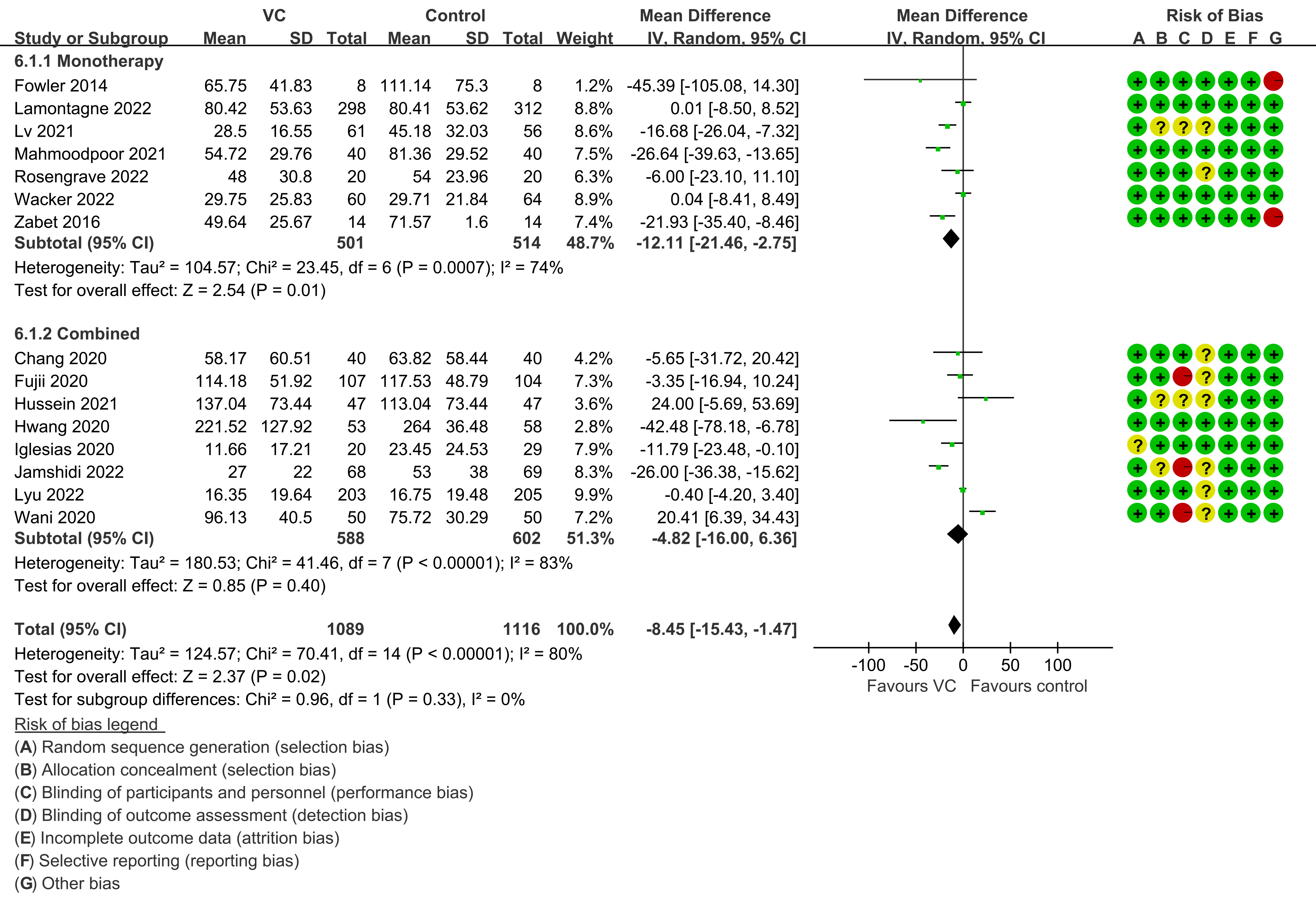

Supplement: Supplementary Figure 1 — Funnel plot assessing the potential publication bias for primary outcomes in septic patients based on IVVC administration. [file Data_Sheet_1.zip › Supplemental Figures/SFig 18.tif]

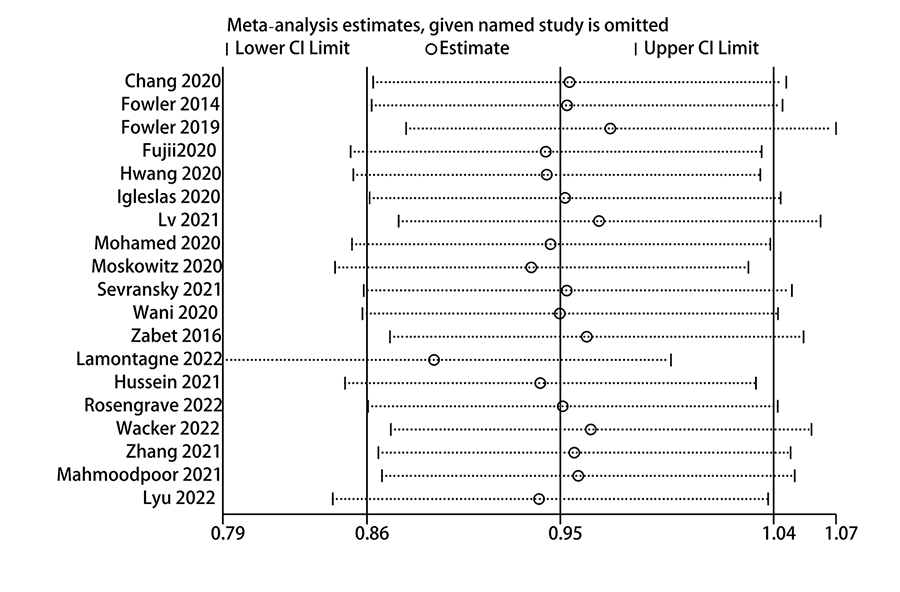

Supplement: Supplementary Figure 1 — Funnel plot assessing the potential publication bias for primary outcomes in septic patients based on IVVC administration. [file Data_Sheet_1.zip › Supplemental Figures/SFig 2.TIF]

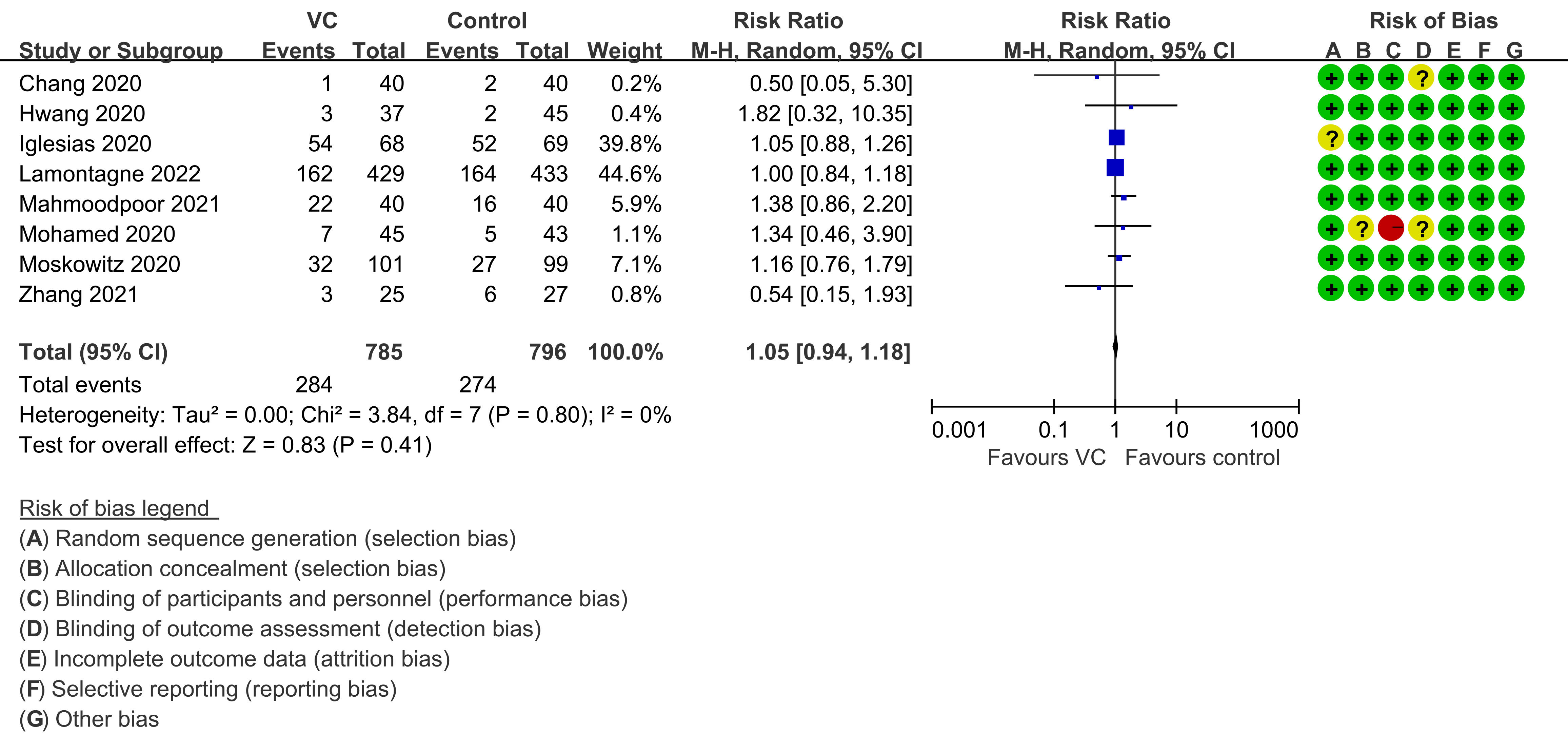

Supplement: Supplementary Figure 1 — Funnel plot assessing the potential publication bias for primary outcomes in septic patients based on IVVC administration. [file Data_Sheet_1.zip › Supplemental Figures/SFig 3.TIF]

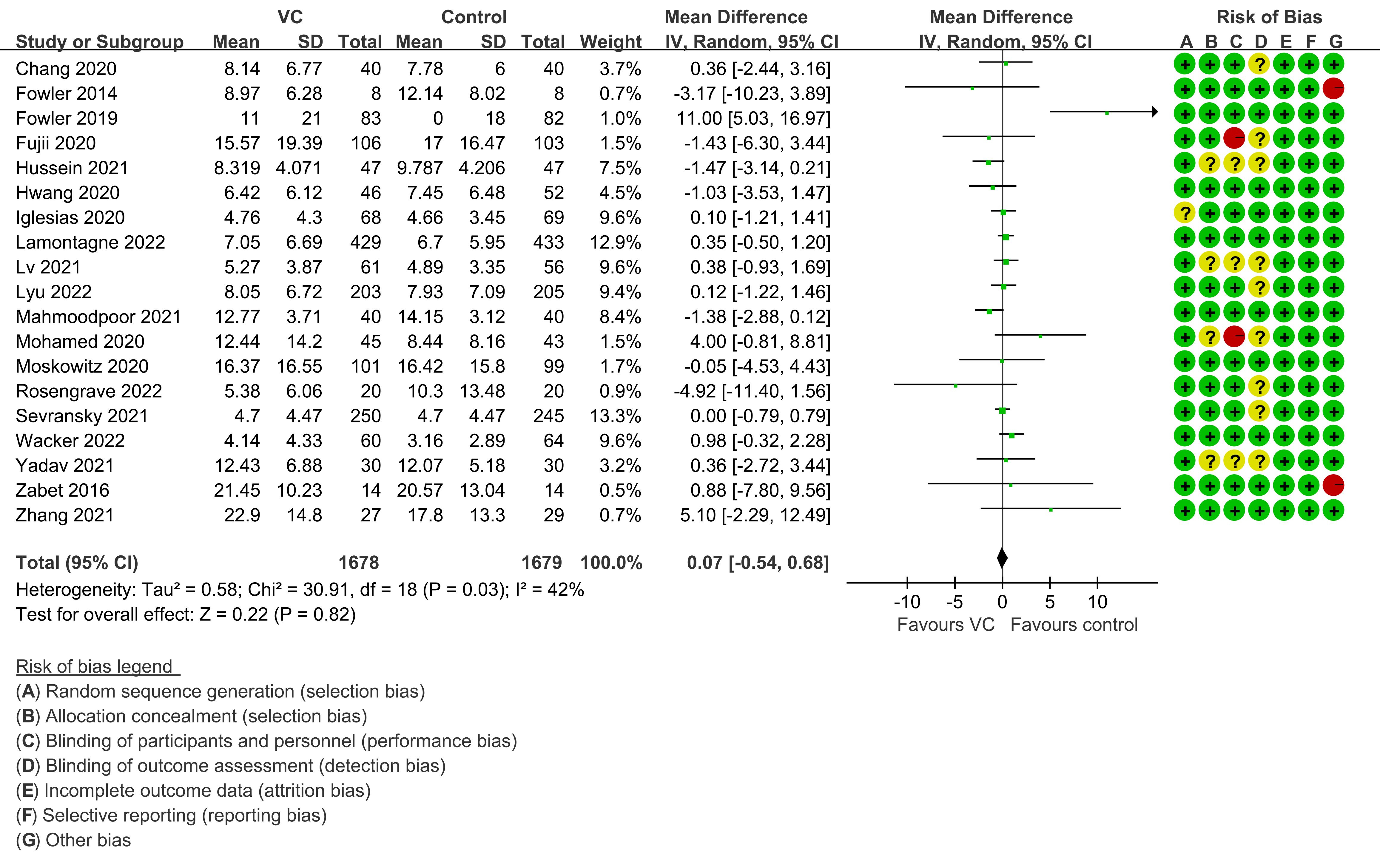

Supplement: Supplementary Figure 1 — Funnel plot assessing the potential publication bias for primary outcomes in septic patients based on IVVC administration. [file Data_Sheet_1.zip › Supplemental Figures/SFig 4.TIF]

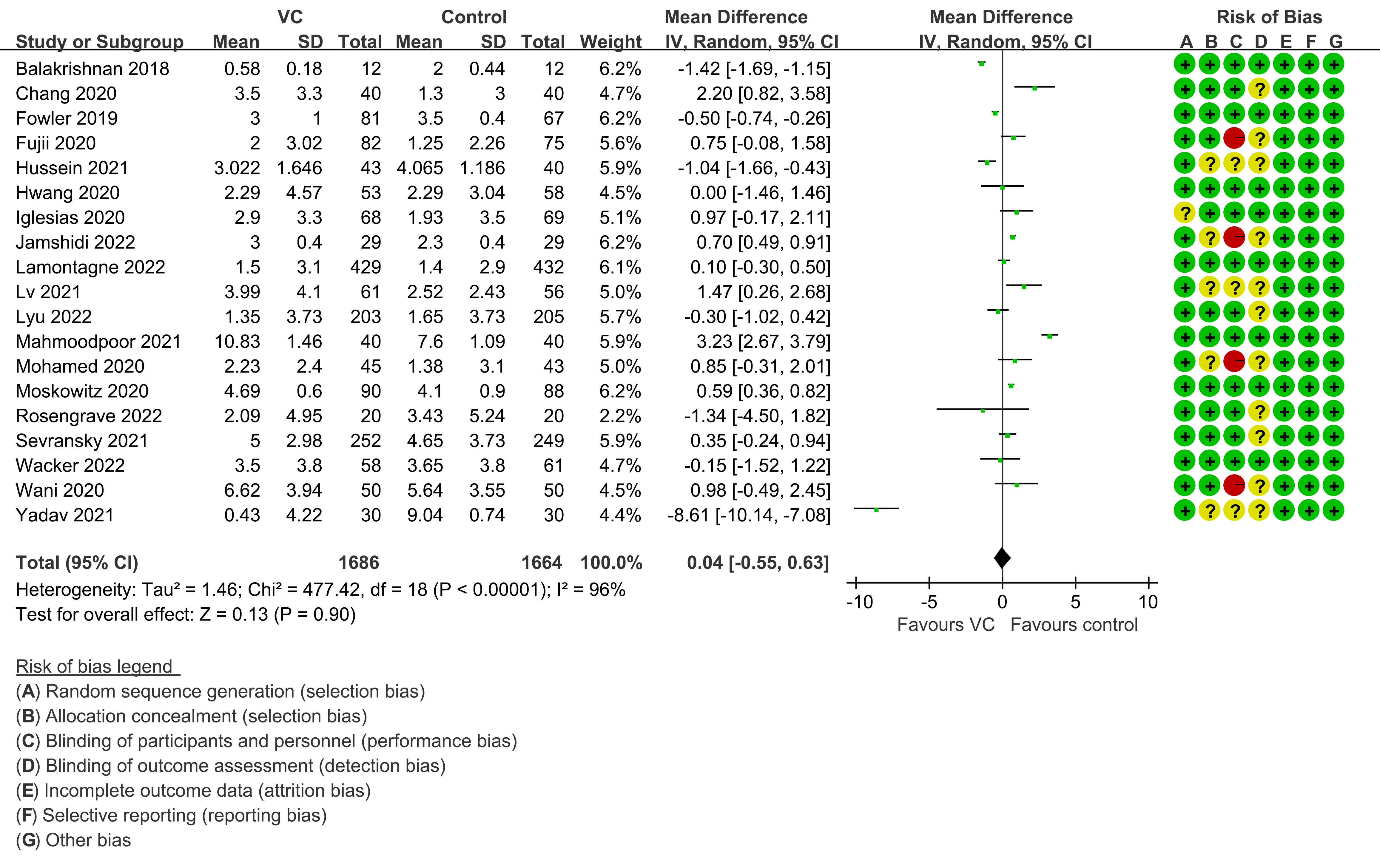

Supplement: Supplementary Figure 1 — Funnel plot assessing the potential publication bias for primary outcomes in septic patients based on IVVC administration. [file Data_Sheet_1.zip › Supplemental Figures/SFig 5.TIF]

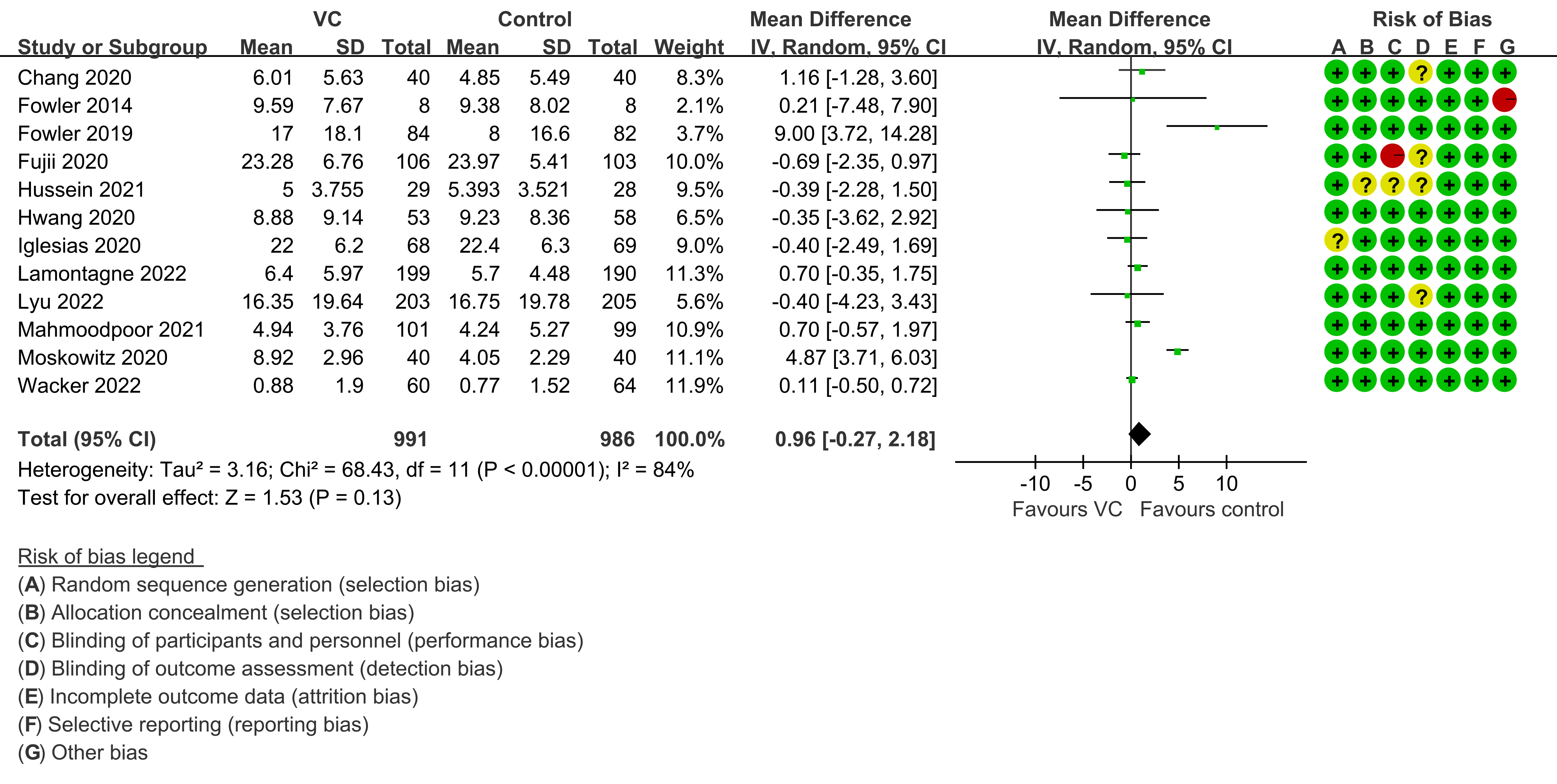

Supplement: Supplementary Figure 1 — Funnel plot assessing the potential publication bias for primary outcomes in septic patients based on IVVC administration. [file Data_Sheet_1.zip › Supplemental Figures/SFig 6.TIF]

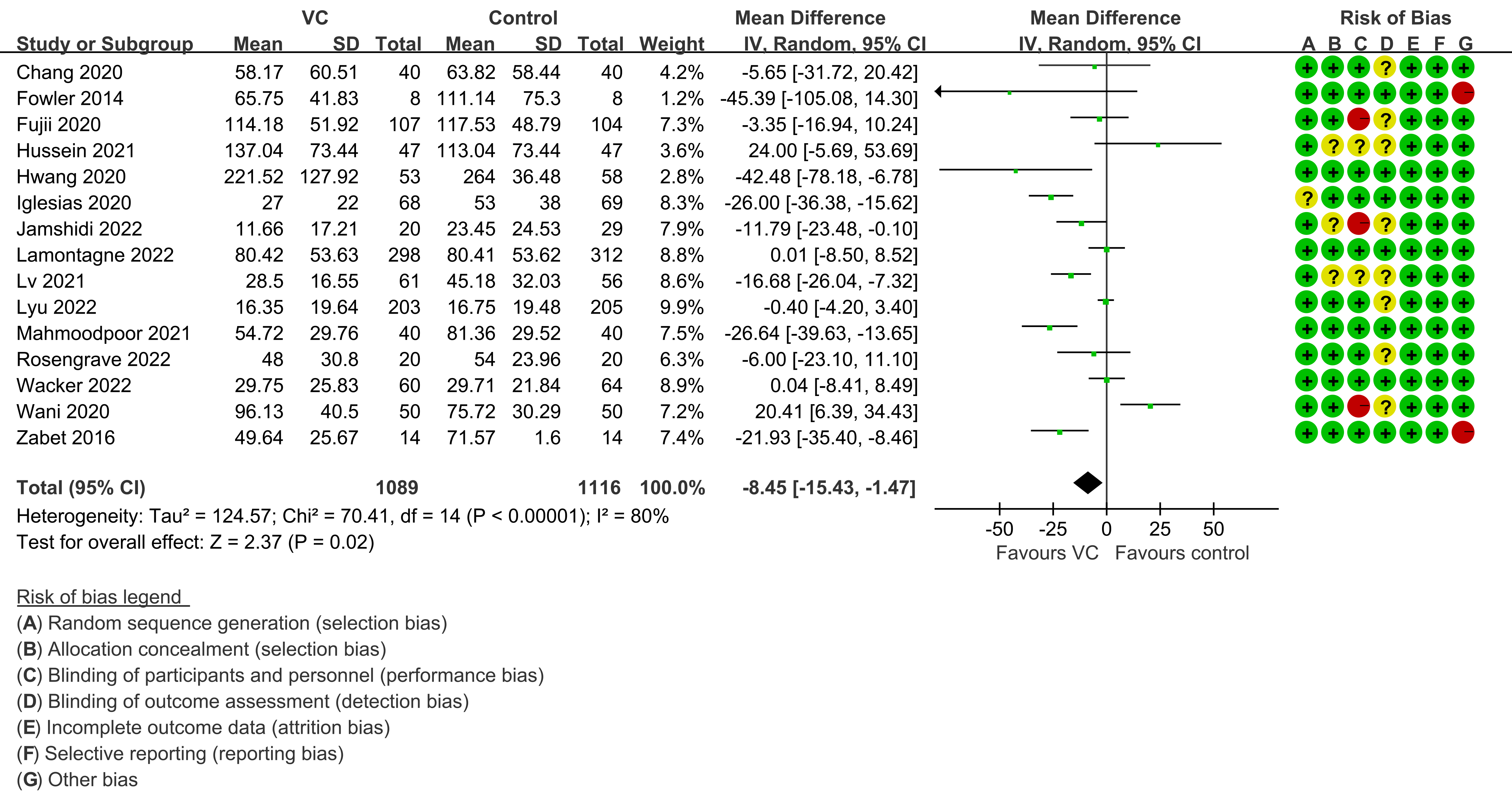

Supplement: Supplementary Figure 1 — Funnel plot assessing the potential publication bias for primary outcomes in septic patients based on IVVC administration. [file Data_Sheet_1.zip › Supplemental Figures/SFig 7.TIF]

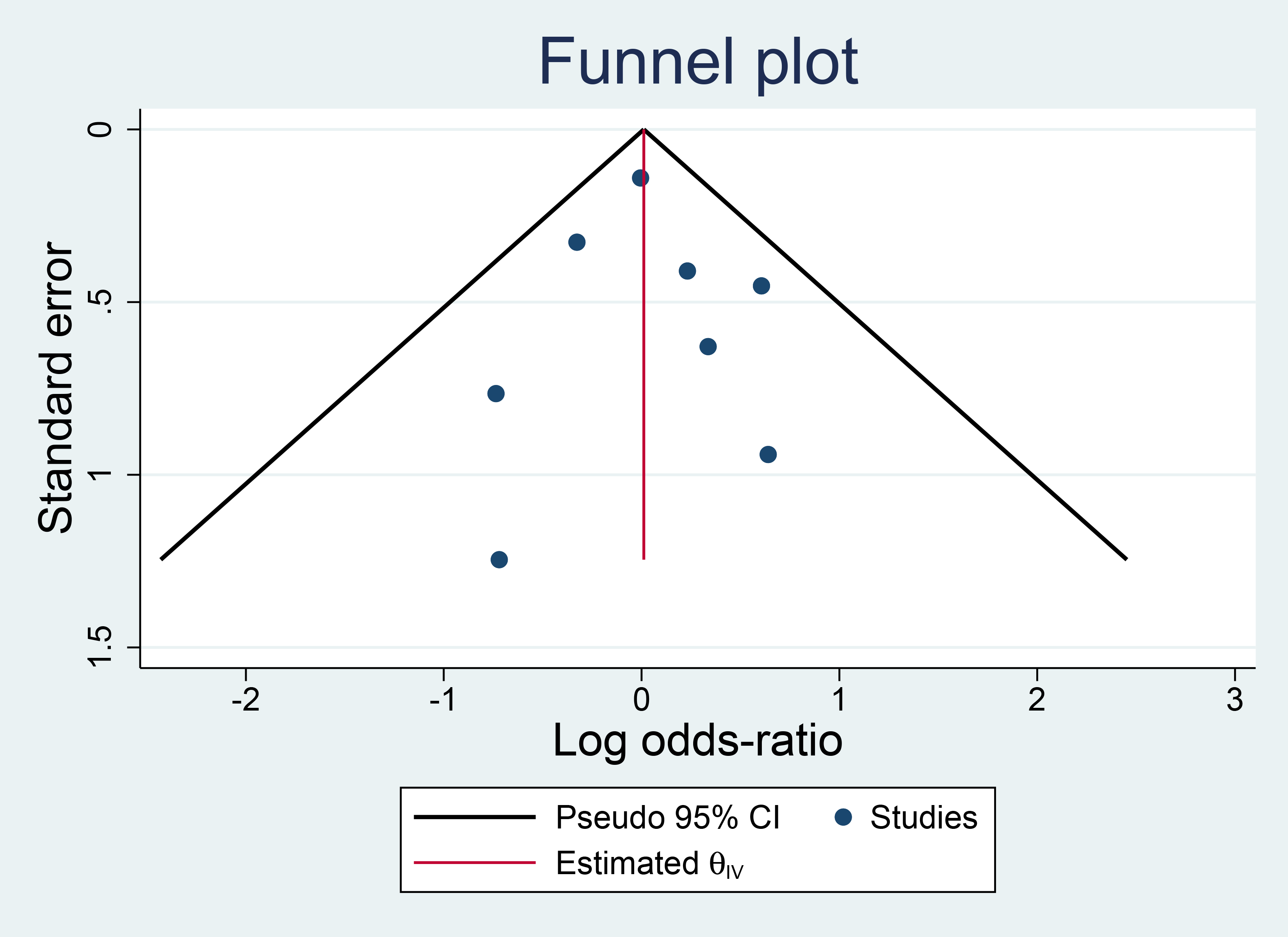

Supplement: Supplementary Figure 1 — Funnel plot assessing the potential publication bias for primary outcomes in septic patients based on IVVC administration. [file Data_Sheet_1.zip › Supplemental Figures/SFig 8.TIF]

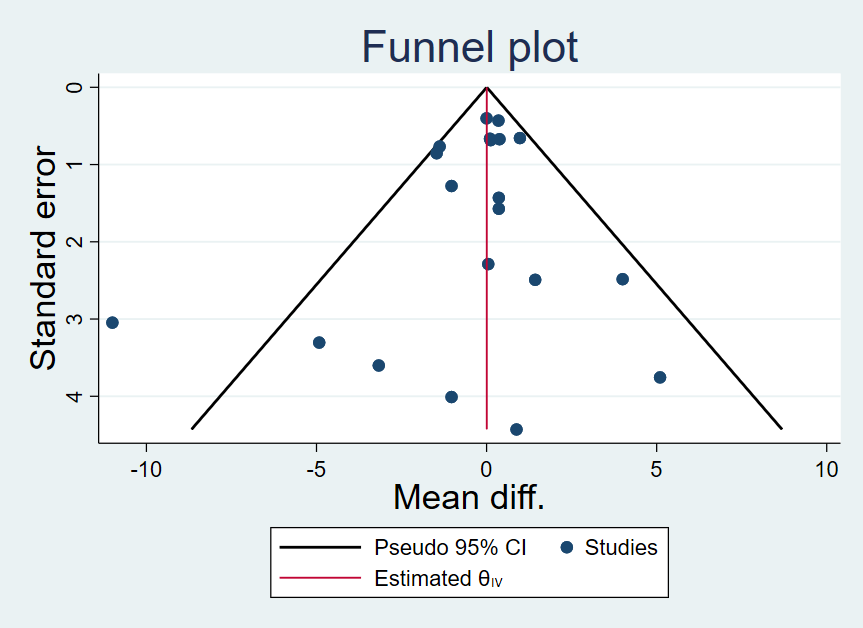

Supplement: Supplementary Figure 1 — Funnel plot assessing the potential publication bias for primary outcomes in septic patients based on IVVC administration. [file Data_Sheet_1.zip › Supplemental Figures/SFig 9.TIF]

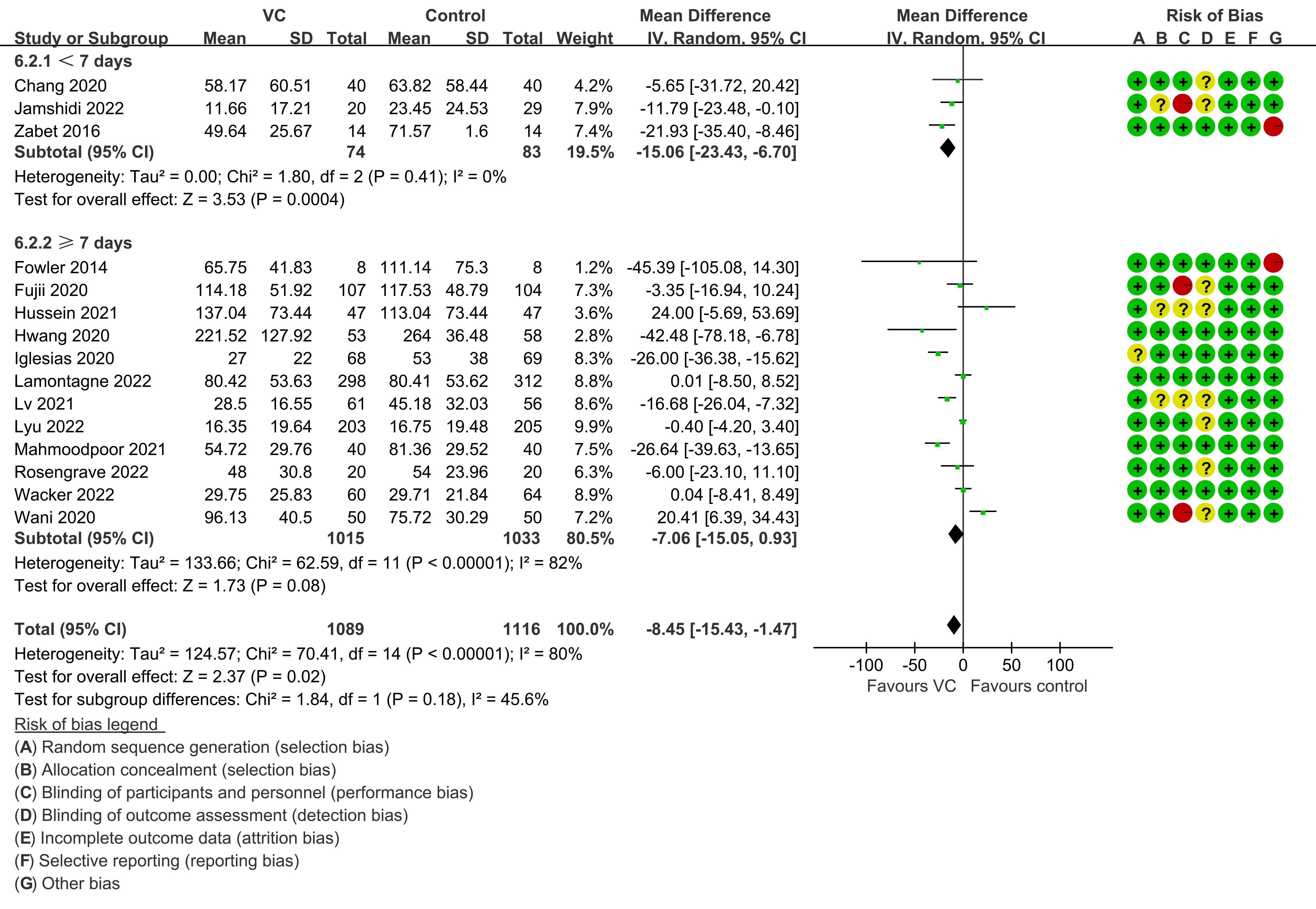

Supplement: Supplementary Figure 1 — Funnel plot assessing the potential publication bias for primary outcomes in septic patients based on IVVC administration. [file Data_Sheet_1.zip › Supplemental Figures/SFig19.tif]
